# Correction: Mycobiome of the Bat White Nose Syndrome Affected Caves and Mines Reveals Diversity of Fungi and Local Adaptation by the Fungal Pathogen *Pseudogymnoascus* (*Geomyce*s) *destructans*

**DOI:** 10.1371/journal.pone.0116149

**Published:** 2014-12-16

**Authors:** The PLOS ONE Staff

Figures 1 and 3 are incorrect. Please view the corrected Figures 1 and 3, as well as the corrected corresponding figure legends, here.

**Figure 1:**
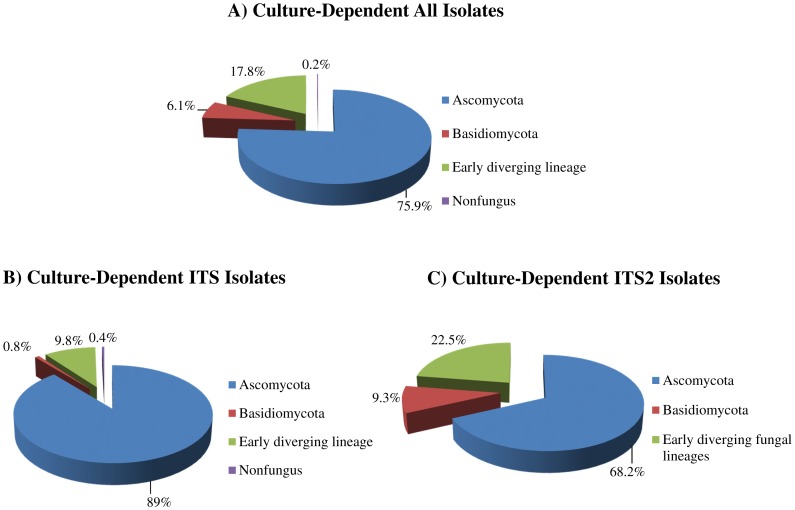
Fungal isolates recovered by culture-dependent methods. (A) Relative distribution of all cultures according to different fungal phyla; (B) Relative distribution of isolates identified by homologies to ITS sequences; (C) Relative distribution of isolates identified by homologies to ITS2 sequences.

**Figure 3:**
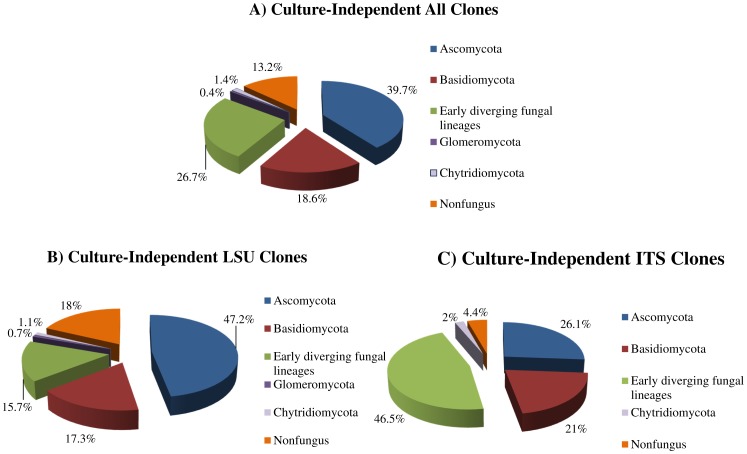
Phyla distribution in culture-independent (CI) clones: A) Relative proportions of different phyla of all clones; B) Relative proportions of different phyla of LSU clones; C) Relative proportions of different phyla of ITS clones.
