# Supplementary figures and images for: Mycobiome of the Bat White Nose Syndrome Affected Caves and Mines Reveals Diversity of Fungi and Local Adaptation by the Fungal Pathogen Pseudogymnoascus (Geomyces) destructans
Source: PLoS One. 2014 Sep 29;9(9):e108714. doi: 10.1371/journal.pone.0108714 (PMC4181696; doi:10.1371/journal.pone.0108714)

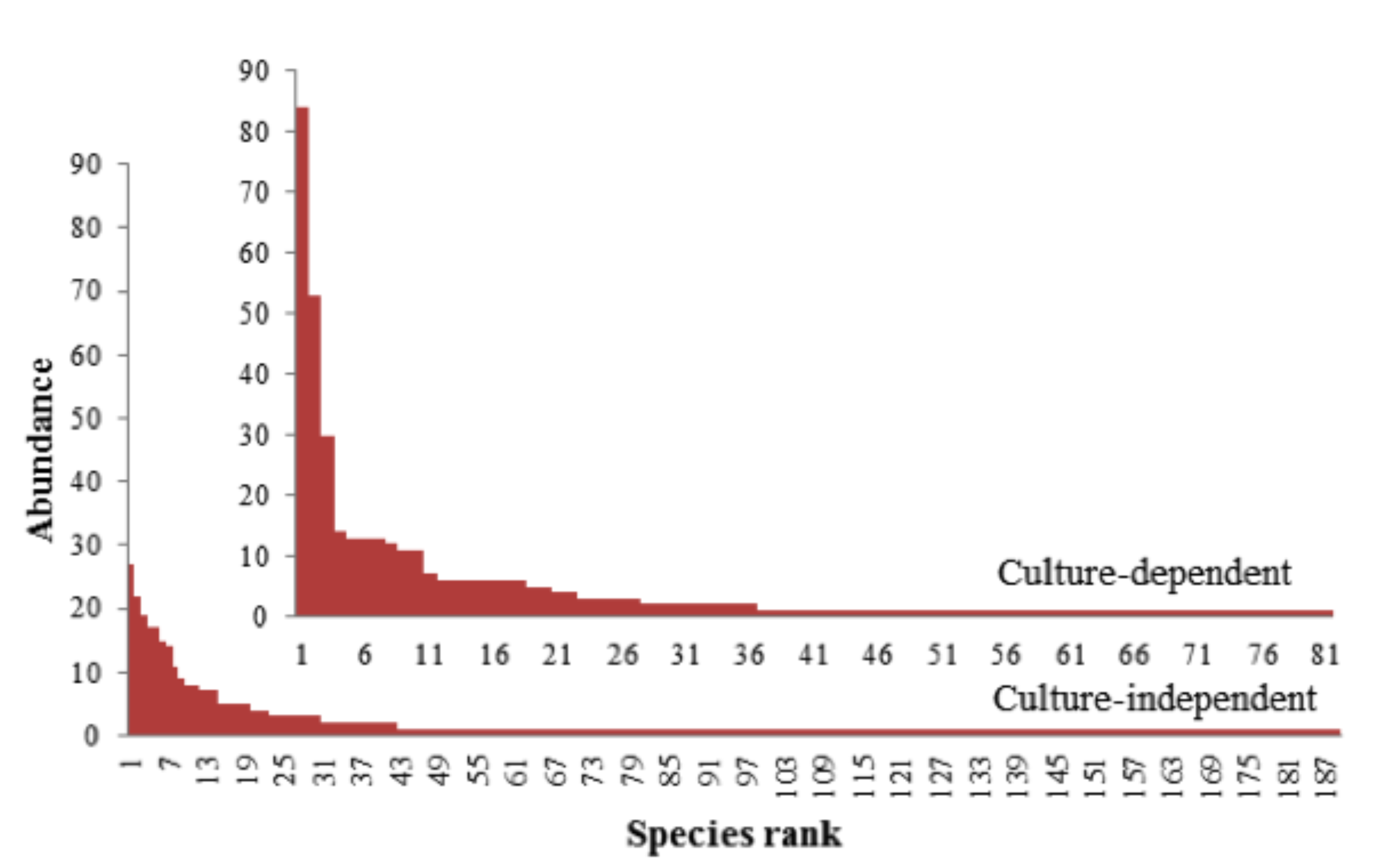

Supplement: Figure S1 — Rank-abundance plots for the culture-dependent and -independent investigations. Abundance of each OTU was indicated by the number of isolates in the culture-dependent method (51 OTUs were singletons in 73 ITS OTUs) or the number of clones in the culture-independent method (167 OTUs were singletons in 189 LUS OTUs). (TIF) [file pone.0108714.s001.tif]
