# Supplementary material for: Mycobiome of the Bat White Nose Syndrome Affected Caves and Mines Reveals Diversity of Fungi and Local Adaptation by the Fungal Pathogen Pseudogymnoascus (Geomyces) destructans
Source: PLoS One. 2014 Sep 29;9(9):e108714. doi: 10.1371/journal.pone.0108714 (PMC4181696; doi:10.1371/journal.pone.0108714)
Supplement: Table S1 — Bat hibernacula in the eastern United States surveyed for Pd by rt-PCR and CD methods. (DOCX) [file pone.0108714.s002.docx]

Table S1. Bat hibernacula in the eastern United States surveyed for *Pd* by rt-PCR and CD methods

| **Number** | **Source** | **Sample**  **Collection** | **State** | **Sample Type** | ***Pd* by rt-PCR** | ***Pd* by CD** |
| --- | --- | --- | --- | --- | --- | --- |
| 38249 | Aeolus Cave#1, E. Dorset | Aug. 2010 | VT | decomposed bat remains | +**^*^** | -**^**^** |
| 38250 | Aeolus Cave #2, E. Dorset | Aug. 2010 | VT | bat remains and debris | + | - |
| 38251 | Aeolus Cave #3, E. Dorset | Aug. 2010 | VT | decomposed bat remains | - | - |
| 38252 | Aeolus Cave #4, E. Dorset | Aug. 2010 | VT | decomposed bat remains | + | - |
| 38253 | Aeolus Cave #5, E. Dorset | Aug. 2010 | VT | decomposed bat remains | + | - |
| 38254 | Aeolus Cave #6, E. Dorset | Aug. 2010 | VT | decomposed bat remains | + | - |
| 38255 | Aeolus Cave #7, E. Dorset | Aug. 2010 | VT | decomposed bat remains | + | - |
| 38257 | Aeolus Cave #8, E. Dorset | Aug. 2010 | VT | sediment under bat remains | + | + |
| 38258 | Aeolus Cave #9, E. Dorset | Aug. 2010 | VT | surface swab | + | - |
| 38259 | Aeolus Cave #10, E. Dorset | Aug. 2010 | VT | surface swab | + | - |
| 38261 | Aeolus Cave #11, E. Dorset | Aug. 2010 | VT | surface swab | - | - |
| 38263 | Aeolus Cave #12, E. Dorset | Aug. 2010 | VT | surface swab | + | - |
| 38264 | Aeolus Cave #13, E. Dorset | Aug. 2010 | VT | decomposed bat remains | + | - |
| 38266 | Aeolus Cave #14, E. Dorset | Aug. 2010 | VT | sediment under bat remains | + | - |
| 38268 | Aeolus Cave #15, E. Dorset | Aug. 2010 | VT | surface swab | + | - |
| 38270 | Hitchcock Mine #1, Paradox, Essex | Jan. 2010 | NY | surf sediment and bat feces | + | - |
| 38273 | Hitchcock Mine #2, Paradox, Essex | Jan. 2010 | NY | surface sediment | + | - |
| 38274 | Barton Mine #1, Mineville, Essex | Feb. 2010 | NY | surface sediment | - | - |
| 38275 | Barton Mine #2, Mineville, Essex | Feb. 2010 | NY | surface sediment | + | - |
| 39141 | Williams Preserve Mine, Kinston, Ulster | Nov. 2010 | NY | air | ND | + |
| 39142 | Williams Preserve Mine, Kinston, Ulster | Nov. 2010 | NY | air | ND | - |
| 39143 | Williams Preserve Mine, Kinston, Ulster | Nov. 2010 | NY | air | ND | - |
| 39144 | Williams Preserve Mine, Kinston, Ulster | Nov. 2010 | NY | air | ND | - |
| 39145 | Williams Preserve Mine, Kinston, Ulster | Nov. 2010 | NY | swab | - | + |
| 39147 | Williams Preserve Mine, Kinston, Ulster | Nov. 2010 | NY | swab | - | + |
| 39148 | Williams Preserve Mine, Kinston, Ulster | Nov. 2010 | NY | swab | - | - |
| 39149 | Williams Preserve Mine, Kinston, Ulster | Nov. 2010 | NY | swab | - | - |
| 39150 | Williams Preserve Mine, Kinston, Ulster | Nov. 2010 | NY | soil | - | - |
| 39151 | Williams Preserve Mine, Kinston, Ulster | Nov. 2010 | NY | soil | - | - |
| 41544 | Williams Preserve Mine WP#1, Ulster | Nov. 2010 | NY | swab | - | + |
| 41546 | Williams Preserve Mine WP#2, Ulster | Nov. 2010 | NY | swab | - | - |
| 41547 | Williams Preserve Mine WP3A, Ulster | Nov. 2010 | NY | swab | + | + |
| 41548 | Williams Preserve Mine WP3B, Ulster | Nov. 2010 | NY | swab | + | + |
| 41549 | Williams Preserve Mine WP#4, Ulster | Nov. 2010 | NY | swab | - | - |
| 41551 | Williams Preserve Mine WP#6, Ulster | Nov. 2010 | NY | swab | - | + |
| 41552 | Williams Preserve Mine WP#7, Ulster | Nov. 2010 | NY | swab | - | - |
| 41553 | Williams Preserve Mine WP#8, Ulster | Nov. 2010 | NY | swab | + | - |
| 41554 | Williams Preserve Mine WP#9, Ulster | Nov. 2010 | NY | swab | - | - |
| 41555 | Williams Preserve Mine WP#3S, Ulster | Nov. 2010 | NY | sediment | - | - |
| 41556 | Williams Preserve Mine WP#4S, Ulster | Nov. 2010 | NY | sediment | - | + |
| 41557 | Williams Preserve Mine WP#5S, Ulster | Nov. 2010 | NY | sediment | - | - |
| 41558 | Williams Preserve Mine WP#6S, Ulster | Nov. 2010 | NY | sediment | - | + |
| 41559 | Williams Preserve Mine WP#8S, Ulster | Nov. 2010 | NY | swab | - | - |
| 41560 | Williams Preserve Mine WP#9S, Ulster | Nov. 2010 | NY | sediment | - | - |
| 6717 | Graphite Mine #1, Hague, Warren, NY | Jan. 2011 | NY | swab | + | - |
| 6719 | Graphite Mine #3, Hague, Warren, NY | Jan. 2011 | NY | swab | - | - |
| 6720 | Graphite Mine #5, Hague, Warren, NY | Jan. 2011 | NY | swab | + | - |
| 6724 | Graphite Mine #6, Hague, Warren, NY | Jan. 2011 | NY | sediment | + | + |
| 6727 | Graphite Mine #8, Hague, Warren, NY | Jan. 2011 | NY | swab | + | - |
| 6728 | Graphite Mine #10, Hague, Warren, NY | Jan. 2011 | NY | swab | - | - |
| 6730 | Graphite Mine #12, Hague, Warren, NY | Jan. 2011 | NY | swab | - | - |
| 6732 | Graphite Mine #15, Hague, Warren, NY | Jan. 2011 | NY | sediment | - | - |
| 6733 | Graphite Mine #17, Hague, Warren, NY | Jan. 2011 | NY | sed/swab? | + | - |
| 6734 | Graphite Mine #19, Hague, Warren, NY | Jan. 2011 | NY | swab | + | + |
| 6743 | Graphite Mine #20, Hague, Warren, NY | Jan. 2011 | NY | sediment | + | - |
| 6745 | Graphite Mine #23, Hague, Warren, NY | Jan. 2011 | NY | swab | + | + |
| 6747 | Graphite Mine #25, Hague, Warren, NY | Jan. 2011 | NY | swab | - | - |
| 6757 | Graphite Mine #26, Hague, Warren, NY | Jan. 2011 | NY | swab | - | + |
| 6759 | Graphite Mine #27, Hague, Warren, NY | Jan. 2011 | NY | sediment | - | - |
| 6762 | Graphite Mine #29, Hague, Warren, NY | Jan. 2011 | NY | sediment | - | - |
| 6763 | Graphite Mine #30, Hague, Warren, NY | Jan. 2011 | NY | swab | + | + |
| 6765 | Graphite Mine #32, Hague, Warren, NY | Jan. 2011 | NY | sediment | + | + |
| 6767 | Graphite Mine #33, Hague, Warren, NY | Jan. 2011 | NY | sediment | - | - |
| 6777 | Barton Mine #1, Moriah, Essex | Feb. 2011 | NY | sediment | - | - |
| 6785 | Barton Mine #3, Moriah, Essex | Feb. 2011 | NY | sediment | + | - |
| 6786 | Barton Mine #5, Moriah, Essex | Feb. 2011 | NY | sediment | - | - |
| 6789 | Barton Mine #6, Moriah, Essex | Feb. 2011 | NY | swab | + | - |
| 6790 | Barton Mine #7, Moriah, Essex | Feb. 2011 | NY | swab | - | - |
| 6791 | Barton Mine #10, Moriah, Essex | Feb. 2011 | NY | sediment | - | - |
| 6792 | Barton Mine #11, Moriah, Essex | Feb. 2011 | NY | sediment | + | - |
| 6793 | Barton Mine #12, Moriah, Essex | Feb. 2011 | NY | swab | + | - |
| 6794 | Barton Mine #14, Moriah, Essex | Feb. 2011 | NY | swab | - | - |
| 6795 | Barton Mine #18, Moriah, Essex | Feb. 2011 | NY | swab | + | - |
| 6796 | Barton Mine #22, Moriah, Essex | Feb. 2011 | NY | swab | + | - |
| 6797 | Barton Mine #23, Moriah, Essex | Feb. 2011 | NY | sediment | - | - |
| 6798 | Barton Mine #25, Moriah, Essex | Feb. 2011 | NY | sediment | + | - |
| 6799 | Barton Mine #27, Moriah, Essex | Feb. 2011 | NY | swab | - | - |
| 6801 | Barton Mine #28, Moriah, Essex | Feb. 2011 | NY | swab | + | - |
| 6802 | Barton Mine #31, Moriah, Essex | Feb. 2011 | NY | sediment | - | - |
| 6803 | Barton Mine #33, Moriah, Essex | Feb. 2011 | NY | sediment | - | - |
| 6804 | Barton Mine #39, Moriah, Essex | Feb. 2011 | NY | sediment | - | - |
| 6805 | Hailes Cave #1, Guilderland, Albany | Feb. 2011 | NY | sediment | - | - |
| 6806 | Hailes Cave #2, Guilderland, Albany | Feb. 2011 | NY | swab | - | - |
| 6807 | Hailes Cave #4, Guilderland, Albany | Feb. 2011 | NY | swab | + | - |
| 6808 | Hailes Cave #5, Guilderland, Albany | Feb. 2011 | NY | swab | - | - |
| 6810 | Hailes Cave #6, Guilderland, Albany | Feb. 2011 | NY | swab | + | - |
| 6811 | Hailes Cave #9, Guilderland, Albany | Feb. 2011 | NY | swab | + | - |
| 6812 | Hailes Cave #10, Guilderland, Albany | Feb. 2011 | NY | swab | + | - |
| 6814 | Hailes Cave #14, Guilderland, Albany | Feb. 2011 | NY | swab | - | - |
| 6817 | Hailes Cave #22, Guilderland, Albany | Feb. 2011 | NY | Dry feces/sediment (racoon?) | - | - |

Note: “?”, indicates the type of sample was not clear. ^*^ denotes positive (+) or negative (-) for *Pd* gDNA; ^**^ denotes positive (+) or negative (-) for *Pd* culture; CD, culture-dependent; rt-PCR, real-time PCR; ND, not done
