# Supplementary material for: Mycobiome of the Bat White Nose Syndrome Affected Caves and Mines Reveals Diversity of Fungi and Local Adaptation by the Fungal Pathogen Pseudogymnoascus (Geomyces) destructans
Source: PLoS One. 2014 Sep 29;9(9):e108714. doi: 10.1371/journal.pone.0108714 (PMC4181696; doi:10.1371/journal.pone.0108714)
Supplement: Table S2 — Environmental samples used for library construction. (DOCX) [file pone.0108714.s003.docx]

Table S2. Environmental samples used for library construction

| **Source** | **Sample type**  **(Sample no.)** | **LSU clone** | **ITS clone** | ***Pd* real-PCR screening** |
| --- | --- | --- | --- | --- |
| Graphite mine Warren, NY (GMW) | Sediment (6724, 6743) | GMW_D01 | GMW_S01 | + |
|  | Swab  (6734, 6745) | GMW_D06 | GMW_S06 | + |
| Barton mine, Essex, NY (BME) | Sediment (6785, 6792) | BME_D02 | BME_S02 | + |
|  | Swab  (6789, 6801) | BME_D07 | BME_S07 | + |
|  | Swab  (38274, 38275) | BME_D010 | BME_S10 | -/+ |
| Hailes cave, Albany, NY (HCA) | Sediment (6805, 6817) | HCA_D03 | HCA_S03 | - |
|  | Swab  (6807, 6811) | HCA_D08 | HCA_S08 | + |
| Aeolus cave, Bennington, VT (ACV) | Sediment (38257, 38266) | ACV_D04 | ACV_S04 | + |
|  | Swab  (38258, 38263) | ACV_D09 | ACV_S09 | + |
| Williams mine, Ulster, NY (WMU) | Sediment (39150, 39151) | WMU_D011 | WMU_S11 | - |
|  | Swab  (39145, 41549) | WMU_D012 | WMU_S12 | -/+ |
| Hitchcock mine, Essex, NY (HME) | Sediment (38270, 38273) | HME_D05 | HME_S05 | + |

Note: The sample number in parenthesis under the sample type column are same source as described in table S1; positive (+) or negative (-) for presence or absence of *Pd* gDNA
