# Supplementary material for: Mycobiome of the Bat White Nose Syndrome Affected Caves and Mines Reveals Diversity of Fungi and Local Adaptation by the Fungal Pathogen Pseudogymnoascus (Geomyces) destructans
Source: PLoS One. 2014 Sep 29;9(9):e108714. doi: 10.1371/journal.pone.0108714 (PMC4181696; doi:10.1371/journal.pone.0108714)
Supplement: Table S3 — GenBank accession numbers of ribosomal gene sequences by CD and CI methods. (DOCX) [file pone.0108714.s004.docx]

Table S3. GenBank accession numbers of ribosomal gene sequences by CD and CI methods

| **Cultures** | **Category** | **Parameters** | **Numbers** | **Accession numbers** |
| --- | --- | --- | --- | --- |
|  | ITS | ITS2 | 399 | KC008730-KC009128 |
|  |  | ITS | 237 | KC009286-KC009522 |
|  | LSU | LSU | 39 | KC009247-KC009285 |
| **Culture-independent clones** | LSU | Fungal confirmed sequences | 330 | JX534602-JX534931 |
|  |  | Fungal sequences | 40 | JX898636-JX898675 |
|  |  | Non fungal sequences | 62 | JX545249-JX545310 |
|  |  | Chimera sequences non fungal | 19 | JX898533-JX898551 |
|  | ITS | 100 % coverage sequences | 168 | JX675050-JX675217 |
|  |  | 50-99% coverage sequences | 55 | JX898552-JX898606 |
|  |  | <50% coverage sequences | 18 | JX898607-JX898624 |
|  |  | Non fungal sequences | 11 | JX898625-JX898635 |
