# Supplementary material for: Mycobiome of the Bat White Nose Syndrome Affected Caves and Mines Reveals Diversity of Fungi and Local Adaptation by the Fungal Pathogen Pseudogymnoascus (Geomyces) destructans
Source: PLoS One. 2014 Sep 29;9(9):e108714. doi: 10.1371/journal.pone.0108714 (PMC4181696; doi:10.1371/journal.pone.0108714)
Supplement: Table S4 — Details of ITS2 sequences of fungal isolates recovered by CD method. (DOCX) [file pone.0108714.s005.docx]

Table S4. Details of ITS2 sequences of fungal isolates recovered by CD method

| Sum  (%)^a^ | OTU^b^ | Accession no. | Best BLAST hit  Taxon Phylum Class Score^c^ Acc. no.^d^ | | | | | %Similiarity^e^ |
| --- | --- | --- | --- | --- | --- | --- | --- | --- |
| 0.42 | 6801-S7 | KC009061 | *Mucor hiemalis* | EDFL | Zygomycetes | 366 | JX845571 | 100 |
| 0.42 | *6801-S6 | KC009493 | *Verticillium leptobactrum* | Ascomycota | Deuteromycetes | 592 | EF641874 | 98 |
| 0.42 | *6801-R1 | KC009494 | *Mortierella polycephala* | EDFL | Zygomycetes | 711 | HQ630334 | 98 |
| 0.42 | 6803-S3 | KC009497 | *Verticillium* sp. | Ascomycota | Deuteromycetes | 443 | DQ888742 | 99 |
| 0.42 | 6805-S6 | KC009499 | *Podospora* sp. | Ascomycota | Sordariomycetes | 500 | JN689974 | 99 |
| 0.42 | 6806-R2 | KC009503 | *Kernia* sp. | Ascomycota | Sordariomycetes | 607 | FJ946487 | 98 |
| 0.42 | 6807-R1 | KC009508 | *Mortierella* sp. | EDFL | Zygomycetes | 731 | HQ630307 | 100 |
| 0.42 | 39143-R3 | KC009347 | *Mortierella* sp. | EDFL | Zygomycetes | 737 | EU240130 | 99 |
| 0.42 | *39144-S5 | KC009348 | *Penicillium brevicompactum* | Ascomycota | Eurotiomycetes | 625 | AY373898 | 99 |
| 0.42 | 39148-R5 | KC009356 | *Cosmospora* sp. | Ascomycota | Sordariomycetes | 614 | JN995627 | 99 |
| 0.42 | 39149-S1 | KC009358 | *Podospora* sp. | Ascomycota | Sordariomycetes | 601 | HQ647346 | 99 |
| 0.42 | 6808-S2 | KC009510 | *Aspergillus asperescens* | Ascomycota | Eurotiomycetes | 61 | EF652475 | 100 |
| 0.42 | *38273-S5 | KC009332 | *Mortierella parvispora* | EDFL | Zygomycetes | 737 | EU484279 | 99 |
| 0.42 | *38273-R2 | KC009334 | *Chaetomium crispatum* | Ascomycota | Sordariomycetes | 612 | HM365267 | 100 |
| 0.42 | 38275-R1 | KC009339 | *Gymnoascus* sp. | Ascomycota | Eurotiomycetes | 352 | AB361643 | 94 |
| 0.42 | 6759-S3 | KC009452 | *Hypomyces aurantius* | Ascomycota | Sordariomycetes | 482 | AB591044 | 99 |
| 0.42 | *6759-R2 | KC009454 | *Mucor flavus* | EDFL | Zygomycetes | 637 | EU484282 | 98 |
| 0.42 | 6762-S5 | KC009462 | *Chrysosporium* sp. | Ascomycota | Eurotiomycetes | 556 | AM949568 | 95 |
| 0.42 | 6762-R6 | KC009466 | *Wardomyces humicola* | Ascomycota | Sordariomycetes | 590 | AM774157 | 97 |
| 0.42 | *6763-S5 | KC009467 | *Penicillium swiecickii* | Ascomycota | Eurotiomycetes | 601 | GU441580 | 99 |
| 0.42 | 6765-S5 | KC009469 | *Arthroderma* sp. | Ascomycota | Euascomycetes | 518 | AJ877216 | 92 |
| 0.42 | 6786-R2 | KC009475 | *Mucoromycote* sp. | EDFL | Zygomycetes | 461 | EF555501 | 84 |
| 0.42 | 39141-R2 | KC009343 | *Penicillium polonicum* | Ascomycota | Eurotiomycetes | 540 | JN368451 | 100 |
| 0.42 | 41554-4 | KC009372 | *Mortierella turficola* | EDFL | Zygomycetes | 614 | HQ630350 | 97 |
| 0.42 | 6724-S2 | KC009391 | *Simplicillium* sp. | Ascomycota | Sordariomycetes | 590 | AB604004 | 97 |
| 0.42 | *6727-R1 | KC009401 | *Geomyces pannorum* | Ascomycota | Leotiomycetes | 605 | HQ115661 | 100 |
| 0.42 | 6747-S9 | KC009439 | *Debaryomyces hansenii* | Ascomycota | Saccharomycetes | 684 | JQ912667 | 100 |
| 0.42 | 6810-R7 | KC009518 | *Kernia* sp. | Ascomycota | Sordariomycetes | 515 | DQ318208 | 97 |
| 0.42 | 6743-R3 | KC009420 | *Penicillium raphiae* | Ascomycota | Eurotiomycetes | 639 | JN617673 | 100 |
| 0.42 | 6747-S1 | KC009432 | *Penicillium virgatum* | Ascomycota | Eurotiomycetes | 594 | JF439503 | 98 |
| 0.42 | 6747-S4 | KC009435 | *Penicillium angulare* | Ascomycota | Eurotiomycetes | 583 | AY313613 | 97 |
| 0.42 | 6757-R3 | KC009448 | *Mortierella* sp. | EDFL | Zygomycetes | 493 | JQ670951 | 94 |
| 0.42 | 6717-S2 | KC009383 | *Mortierella* sp. | EDFL | Zygomycetes | 682 | AY842393 | 95 |
| 0.84 | 6791-R5 | KC009478 | *Penicillium sanguifluum* | Ascomycota | Eurotiomycetes | 634 | JN617711 | 99 |
| 0.84 | 38257-R4 | KC009302 | *Cordyceps militaris* | Ascomycota | Sordariomycetes | 585 | AF122036 | 97 |
| 0.84 | 38263-S2 | KC009314 | *Verticillium* sp. | Ascomycota | Deuteromycetes | 751 | FJ025166 | 98 |
| 0.84 | 39141-S3 | KC009341 | *Cladosporium cladosporioides* | Ascomycota | Dothideomycetes | 524 | JX077073 | 100 |
| 0.84 | *39143-S1 | KC009345 | *Hypocrea pachybasioides* | Ascomycota | Sordariomycetes | 619 | JX406549 | 100 |
| 0.84 | *38258-S4 | KC009304 | *Mortierella alpina* | EDFL | Zygomycetes | 755 | AB476415 | 100 |
| 0.84 | 6747-S6 | KC009436 | *Isaria farinosa* | Ascomycota | Sordariomycetes | 630 | HQ115724 | 100 |
| 0.84 | *6747-R4 | KC009441 | *Guehomyces pullulans* | Ascomycota | Tremellomycetes | 682 | AF444417 | 99 |
| 0.84 | 41559-2 | KC009379 | *Arachniotus aurantiacus* | Ascomycota | Eurotiomycetes | 610 | HM991267 | 98 |
| 1.26 | 6719-S3 | KC009384 | *Penicillium thomii* | Ascomycota | Eurotiomycetes | 627 | JN585937 | 100 |
| 1.26 | 6759-S1 | KC009450 | *Mortierella* sp. | EDFL | Zygomycetes | 697 | AY157495 | 95 |
| 1.26 | 38257-S2 | KC009300 | *Wardomyces inflatus* | Ascomycota | Sordariomycetes | 666 | HQ914934 | 99 |
| 1.68 | 6730-R4 | KC009411 | *Myxotrichum* sp. | Ascomycota | Leotiomycetes | 542 | AF062815 | 97 |
| 2.10 | *38275-R2 | KC009340 | *Helicostylum pulchrum* | EDFL | Zygomycetes | 704 | AB614353 | 99 |
| 2.10 | 6796-R2 | KC009485 | *Helicostylum* sp. | EDFL | Zygomycetes | 569 | AB614353 | 93 |
| 2.10 | 41557-1 | KC009376 | *Doratomyces stemonitis* | Ascomycota | Sordariomycetes | 518 | JN104543 | 100 |
| 2.10 | 6728-R3 | KC009405 | *Polypaecilum botryoides* | Ascomycota | Eurotiomycetes | 494 | CBS 176.44 | 100 |
| 2.52 | *6720-R1 | KC009389 | *Trichosporon dulcitum* | Ascomycota | Tremellomycetes | 763 | NR_073248 | 100 |
| 2.52 | 6730-R | KC009412 | *Fusarium merismoides* | Ascomycota | Sordariomycetes | 535 | EU860057 | 100 |
| 6.30 | 6743-S4 | KC009418 | *Arthroderma* sp. | Ascomycota | Euascomycetes | 480 | JN104536 | 94 |
| 7.98 | 6810-S1 | KC009515 | *Kernia* sp. | Ascomycota | Sordariomycetes | 502 | DQ318208 | 97 |
| 7.98 | *41549-1 | KC009370 | *Oidiodendron truncatum* | Ascomycota | Leotiomycetes | 592 | FJ914713 | 100 |
| 11.8 | *38270-7 | KC009329 | *Geomyces pannorum* | Ascomycota | Leotiomycetes | 489 | AB517942 | 99 |
| 25.6 | 38259-S4 | KC009307 | *Penicillium polonicum* | Ascomycota | Eurotiomycetes | 634 | JQ082508 | 100 |

^a^Relative abundance for the combined libraries, which was used to sort the entries.

^b^OTUs were characterized by Mothur program [1], the OTU is ≥97% similar to a fungal isolate.

^c^BLASTN [2] score value.

^d^Accession number of the closest database match.

^e^Level of similarity for pairwise alignments with the closest match, using the Martinez-Needleman-Wunsch algorithm [2].

^*^Common OTUs identified from ITS2, ITS, and LSU sequences
