# Supplementary material for: Mycobiome of the Bat White Nose Syndrome Affected Caves and Mines Reveals Diversity of Fungi and Local Adaptation by the Fungal Pathogen Pseudogymnoascus (Geomyces) destructans
Source: PLoS One. 2014 Sep 29;9(9):e108714. doi: 10.1371/journal.pone.0108714 (PMC4181696; doi:10.1371/journal.pone.0108714)
Supplement: Table S5 — Details of ITS sequences of fungal isolates recovered by CD method. (DOCX) [file pone.0108714.s006.docx]

Table S5. Details of ITS sequences of fungal isolates recovered by CD method

| Sum  (%)^a^ | OTU^b^ | Accession no. | Best BLAST hit  Taxon Phylum Class Score^c^ Acc. no.^d^ | | | | | %Similiarity^e^ |
| --- | --- | --- | --- | --- | --- | --- | --- | --- |
| 0.25 | 38249-2 | KC008730 | *Phoma sclerotioides* | Ascomycota | Dothideomycetes | 726 | FJ179161 | 99 |
| 0.25 | 38249-8 | KC008734 | *Mortierella stylospora* | EDFL | Zygomycetes | 507 | JX976086 | 88 |
| 0.25 | 38251-R1 | KC008746 | *Arthroderma silverae* | Ascomycota | Eurotiomycetes | 608 | AJ877216 | 89 |
| 0.25 | 38254-S4 | KC008757 | *Mortierella fimbricystis* | EDFL | Zygomycetes | 904 | JX976069 | 95 |
| 025 | 38253-R3 | KC008754 | *Neonectria ramulariae* | Ascomycota | Sordariomycetes | 854 | AY781232 | 98 |
| 0.25 | 39147-S1 | KC008820 | *Pleosporales sp.* | Ascomycota | Dothideomycetes | 767 | HQ713762 | 99 |
| 0.25 | 39142-S4 | KC008804 | *Dothiorella gregaria* | Ascomycota | Dothideomycetes | 793 | AB470899 | 100 |
| 0.25 | 6777-S7 | KC008970 | *Aphanocladium album* | Ascomycota | Sordariomycetes | 545 | KC291734 | 90 |
| 0.25 | 6805-S1 | KC009082 | *Bionectria ochroleuca* | Ascomycota | Sordariomycetes | 817 | JN002172 | 99 |
| 0.25 | 41559-3 | KC008871 | *Simplicillium lanosoniveum* | Ascomycota | Sordariomycetes | 701 | KC172069 | 92 |
| 0.25 | 6717-S1 | KC008872 | *Verticillium leptobactrum* | Ascomycota | Sordariomycetes | 881 | EF641870 | 99 |
| 0.25 | 6811-S2 | KC009105 | *Beauveria bassiana* | Ascomycota | Sordariomycetes | 872 | KC753398 | 100 |
| 0.25 | 6806-S6 | KC009090 | *Podospora sp.* | Ascomycota | Sordariomycetes | 647 | AY999127 | 93 |
| 0.25 | 39144-S6 | KC008813 | *Leotiomycetes sp.* | Ascomycota | Leotiomycetes | 641 | AB190398 | 99 |
| 0.25 | 39144-S2 | KC008810 | *Candida pseudoglaebosa* | Ascomycota | Saccharomycetales | 915 | NR_111588 | 99 |
| 0.25 | *6811-S1 | KC009104 | *Mortierella alpina* | EDFL | Zygomycetes | 1013 | KF313129 | 100 |
| 0.25 | 41554-2 | KC008862 | *Mortierellales sp.* | EDFL | Zygomycetes | 1033 | [DQ865089](http://www.ncbi.nlm.nih.gov/nucleotide/114704069?report=genbank&log$=nucltop&blast_rank=11&RID=SVW2P2TC01R) | 100 |
| 0.25 | 6806-R5 | KC009093 | *Mortierellaceae sp.* | EDFL | Zygomycetes | 939 | [JX975843](http://www.ncbi.nlm.nih.gov/nucleotide/511783620?report=genbank&log$=nucltop&blast_rank=9&RID=SVW6WK3301R) | 99 |
| 0.25 | 6803-R5 | KC009072 | *Mortierella histoplasmatoides* | EDFL | Zygomycetes | 939 | \|  \| [HQ630309](http://www.ncbi.nlm.nih.gov/nucleotide/325516882?report=genbank&log$=nucltop&blast_rank=2&RID=SVWAXC7P01R) \| \| --- \| --- \| | 99 |
| 0.25 | 39141-S5 | KC008799 | *Trichophyton terrestre* | Ascomycota | Eurotiomycetes | 1016 | \|  \| [EF568097](http://www.ncbi.nlm.nih.gov/nucleotide/148535571?report=genbank&log$=nucltop&blast_rank=12&RID=SVWFVUHS01R) \| \| --- \| --- \| | 99 |
| 0.25 | *6797-R1 | KC009053 | *Mortierella sarnyensis* | EDFL | Zygomycetes | 902 | \|  \| [JF311973](http://www.ncbi.nlm.nih.gov/nucleotide/342674857?report=genbank&log$=nucltop&blast_rank=5&RID=SVWP3D6Z01R) \| \| --- \| --- \| | 97 |
| 0.25 | 38268-S2 | KC008781 | *Mortierella sp.* | EDFL | Zygomycetes | 490 | \|  \| [JX975922](http://www.ncbi.nlm.nih.gov/nucleotide/511783699?report=genbank&log$=nucltop&blast_rank=2&RID=SVWTUW1A01R) \| \| --- \| --- \| | 86 |
| 0.25 | *6786-R3 | KC008990 | *Mortierella polycephala* | EDFL | Zygomycetes | 1029 | \|  \| [JX975900](http://www.ncbi.nlm.nih.gov/nucleotide/511783677?report=genbank&log$=nucltop&blast_rank=2&RID=SVWYN1RV01R) \| \| --- \| --- \| | 100 |
| 0.25 | 41547-1 | KC008844 | *Cryptococcus fragicola* | Basidiomycota | Tremellomycetes | 730 | [AB035588](http://www.ncbi.nlm.nih.gov/nucleotide/17827005?report=genbank&log$=nucltop&blast_rank=2&RID=SVX3R4N801R) | 100 |
| 0.25 | 6794-R4 | KC009032 | [*Holtermanniella watticus*](http://blast.st-va.ncbi.nlm.nih.gov/Blast.cgi#alnHdr_383478663) | Basidiomycota | Tremellomycetes | 870 | \|  \| [JQ857031](http://www.ncbi.nlm.nih.gov/nucleotide/383478663?report=genbank&log$=nucltop&blast_rank=4&RID=SVX9W0G301R) \| \| --- \| --- \| | 100 |
| 0.25 | 39142-S2 | KC008802 | *Mucor luteus* | EDFL | Zygomycetes | 929 | \|  \| [NR_103614](http://www.ncbi.nlm.nih.gov/nucleotide/511801439?report=genbank&log$=nucltop&blast_rank=2&RID=SVXFMJZU01R) \| \| --- \| --- \| | 97 |
| 0.25 | 41549-4 | KC008849 | *Mucor flavus* | EDFL | Zygomycetes | 1026 | [JN206049](http://www.ncbi.nlm.nih.gov/nucleotide/409185451?report=genbank&log$=nucltop&blast_rank=2&RID=SVXMYX2C01R) | 98 |
| 0.25 | *6805-R2 | KC009087 | *Mucor flavus* | EDFL | Zygomycetes | 1110 | \|  \| [JN206061](http://www.ncbi.nlm.nih.gov/nucleotide/409185463?report=genbank&log$=nucltop&blast_rank=2&RID=SVXSDYZM01R) \| \| --- \| --- \| | 99 |
| 0.25 | 41544-1 | KC008832 | *Mortierella fimbricystis* | EDFL | Zygomycetes | 785 | [JX976069](http://www.ncbi.nlm.nih.gov/nucleotide/511783846?report=genbank&log$=nucltop&blast_rank=34&RID=SVYYKP9W01R) | 93 |
| 0.25 | 6815-R3 | KC009121 | *Umbelopsis isabellina* | EDFL | Zygomycetes | 996 | [JF303862](http://www.ncbi.nlm.nih.gov/nucleotide/327244608?report=genbank&log$=nucltop&blast_rank=2&RID=SVZ2FKNX01R) | 99 |
| 0.25 | 41548-3 | KC008848 | [*Leuconeurospora pulcherrima*](http://blast.st-va.ncbi.nlm.nih.gov/Blast.cgi#alnHdr_528889241) | EDFL | Zygomycetes | 900 | \|  \| [KF049206](http://www.ncbi.nlm.nih.gov/nucleotide/528889241?report=genbank&log$=nucltop&blast_rank=10&RID=SVZ68C7C01R) \| \| --- \| --- \| | 99 |
| 0.25 | 6791-R1 | KC009010 | *Penicillium griseofulvum* | Ascomycota | Eurotiomycetes | 867 | [DQ339549](http://www.ncbi.nlm.nih.gov/nucleotide/84873878?report=genbank&log$=nucltop&blast_rank=32&RID=SVZCUSVR01R) | 99 |
| 0.25 | *6717-R1 | KC008874 | *Mortierella hyalina* | EDFL | Zygomycetes | 822 | \|  \| [JX975983](http://www.ncbi.nlm.nih.gov/nucleotide/511783760?report=genbank&log$=nucltop&blast_rank=9&RID=SW09M1XF01R) \| \| --- \| --- \| | 97 |
| 0.25 | *6807-S2 | KC009095 | *Penicillium quercetorum* | Ascomycota | Eurotiomycetes | 819 | [AY443471](http://www.ncbi.nlm.nih.gov/nucleotide/42415757?report=genbank&log$=nucltop&blast_rank=4&RID=SW0G091R01R) | 98 |
| 0.25 | 39143-R1 | KC008808 | *Botryotinia fuckeliana* | Ascomycota | Leotiomycetes | 815 | \|  \| [KF802809](http://www.ncbi.nlm.nih.gov/nucleotide/575526206?report=genbank&log$=nucltop&blast_rank=3&RID=SW0M25X001R) \| \| --- \| --- \| | 99 |
| 0.25 | 6763-S6 | KC008950 | *Oidiodendron tenuissimum* | Ascomycota | Leotiomycetes | 734 | [NR_111036](http://www.ncbi.nlm.nih.gov/nucleotide/597900371?report=genbank&log$=nucltop&blast_rank=28&RID=SW0SA2GM01R) | 95 |
| 0.25 | 6806-S2 | KC009089 | *Penicillium glabrum* | Ascomycota | Leotiomycetes | 883 | \|  \| [JX140803](http://www.ncbi.nlm.nih.gov/nucleotide/402513871?report=genbank&log$=nucltop&blast_rank=2&RID=SW0W46MV01R) \| \| --- \| --- \| | 99 |
| 0.25 | 6789-S4 | KC008996 | *Penicillium miczynskii* | Ascomycota | Leotiomycetes | 915 | [JN617670](http://www.ncbi.nlm.nih.gov/nucleotide/372123126?report=genbank&log$=nucltop&blast_rank=2&RID=SW1B0BA301R) | 100 |
| 0.25 | 6808-S5 | KC009098 | *Oidiodendron sp.* | Ascomycota | Leotiomycetes | 695 | [NR_111036](http://www.ncbi.nlm.nih.gov/nucleotide/597900371?report=genbank&log$=nucltop&blast_rank=31&RID=SW1EC6X401R) | 94 |
| 0.25 | 6765-S8 | KC008957 | [*Penicillium tularense*](http://blast.st-va.ncbi.nlm.nih.gov/Blast.cgi#alnHdr_472833348) | Ascomycota | Leotiomycetes | 806 | [KC427195](http://www.ncbi.nlm.nih.gov/nucleotide/472833348?report=genbank&log$=nucltop&blast_rank=3&RID=SW1HZUEK01R) | 98 |
| 0.25 | 6804-R2 | KC009078 | *Hypocrea viridescens* | Ascomycota | Sordariomycetes | 893 | \|  \| [KJ482546](http://www.ncbi.nlm.nih.gov/nucleotide/625295631?report=genbank&log$=nucltop&blast_rank=2&RID=SW1NY4T301R) \| \| --- \| --- \| | 100 |
| 0.25 | 6719-S4 | KC008877 | *Zalerion varium* | Ascomycota | Sordariomycetes | 832 | \|  \| [KF156329](http://www.ncbi.nlm.nih.gov/nucleotide/515473461?report=genbank&log$=nucltop&blast_rank=3&RID=SW1U9K3F01R) \| \| --- \| --- \| | 100 |
| 0.25 | 6743-R2 | KC008924 | *Neobulgaria sp.* | Ascomycota | Leotiomycetes | 845 | [HM051080](http://www.ncbi.nlm.nih.gov/nucleotide/296785429?report=genbank&log$=nucltop&blast_rank=9&RID=SW1Y4VTM01R) | 98 |
| 0.25 | 41558-2 | KC008869 | *Doratomyces sp.* | Ascomycota | Sordariomycetes | 898 | [FJ914706](http://www.ncbi.nlm.nih.gov/nucleotide/237872432?report=genbank&log$=nucltop&blast_rank=7&RID=SW227VS601R) | 99 |
| 0.25 | 6727-S1 | KC008889 | *Chrysosporium vallenarense* | Ascomycota | Eurotiomycetes | 527 | \|  \| [NR_077139](http://www.ncbi.nlm.nih.gov/nucleotide/452056283?report=genbank&log$=nucltop&blast_rank=32&RID=SW277GJ201R) \| \| --- \| --- \| | 85 |
| 0.5 | 6785-S7 | KC008978 | *Auxarthron umbrinum* | Ascomycota | Eurotiomycetes | 749 | \|  \| [NR_111136](http://www.ncbi.nlm.nih.gov/nucleotide/597900471?report=genbank&log$=nucltop&blast_rank=3&RID=SW2C4WDH01R) \| \| --- \| --- \| | 96 |
| 0.5 | 38250-S5 | KC008742 | *Geomyces pannorum* | Ascomycota | Leotiomycetes | 924 | \|  \| [DQ494320](http://www.ncbi.nlm.nih.gov/nucleotide/94959243?report=genbank&log$=nucltop&blast_rank=9&RID=SW2GCESC01R) \| \| --- \| --- \| | 99 |
| 0.5 | *38273-R2 | KC008788 | *Chaetomium crispatum* | Ascomycota | Sordariomycetes | 811 | [JQ864439](http://www.ncbi.nlm.nih.gov/nucleotide/387821494?report=genbank&log$=nucltop&blast_rank=17&RID=SW2PUK9Y01R) | 98 |
| 0.5 | 39144-S4 | KC008812 | *Penicillium vulpinum* | Ascomycota | Leotiomycetes | 564 | \|  \| [FJ004321](http://www.ncbi.nlm.nih.gov/nucleotide/222092999?report=genbank&log$=nucltop&blast_rank=4&RID=SW2UNDR801R) \| \| --- \| --- \| | 100 |
| 0.5 | 6785-S5 | KC008977 | *Myxotrichum sp.* | Ascomycota | Leotiomycetes | 708 | \|  \| [AF062815](http://www.ncbi.nlm.nih.gov/nucleotide/3687773?report=genbank&log$=nucltop&blast_rank=4&RID=SW2Y3MEC01R) \| \| --- \| --- \| | 94 |
| 0.5 | 6724-S1 | KC008883 | *Chrysosporium sp.* | Ascomycota | Eurotiomycetes | 603 | [HF548536](http://www.ncbi.nlm.nih.gov/nucleotide/571260117?report=genbank&log$=nucltop&blast_rank=6&RID=SW329XT101R) | 87 |
| 0.5 | *38252-S1 | KC008748 | *Kernia pachypleura* | Ascomycota | Sordariomycetes | 725 | [DQ318208](http://www.ncbi.nlm.nih.gov/nucleotide/83779376?report=genbank&log$=nucltop&blast_rank=6&RID=SW35UJ2R01R) | 97 |
| 0.5 | *39141-S1 | KC008796 | *Hypocrea pachybasioides* | Ascomycota | Sordariomycetes | 941 | [FJ860796](http://www.ncbi.nlm.nih.gov/nucleotide/268633436?report=genbank&log$=nucltop&blast_rank=2&RID=SW39XH9001R) | 100 |
| 0.5 | 6817-S4 | KC009125 | *Mucor hiemalis* | EDFL | Zygomycetes | 913 | \|  \| [JX976246](http://www.ncbi.nlm.nih.gov/nucleotide/519789269?report=genbank&log$=nucltop&blast_rank=4&RID=SW3DBHX501R) \| \| --- \| --- \| | 97 |
| 0.75 | 6747-R2 | KC008936 | *Trichosporon shinodae* | Basidiomycota | Tremellomycetes | 730 | [AB180201](http://www.ncbi.nlm.nih.gov/nucleotide/47776294?report=genbank&log$=nucltop&blast_rank=6&RID=SW3H4RF601R) | 99 |
| 0.75 | *6743-S5 | KC008923 | *Guehomyces pullulans* | Basidiomycota | Tremellomycetes | 896 | \|  \| [KC009015](http://www.ncbi.nlm.nih.gov/nucleotide/530252890?report=genbank&log$=nucltop&blast_rank=9&RID=SW3P053T01R) \| \| --- \| --- \| | 99 |
| 0.75 | 39150-R1 | KC008833 | *Mortierella cf. gamsii* | EDFL | Zygomycetes | 976 | [JX975892](http://www.ncbi.nlm.nih.gov/nucleotide/511783669?report=genbank&log$=nucltop&blast_rank=6&RID=SW3WMWTH01R) | 99 |
| 0.75 | 39150-R2 | KC008834 | *Tetracladium sp.* | Ascomycota | Mitosporic Ascomycota | 826 | [KC180672](http://www.ncbi.nlm.nih.gov/nucleotide/443500491?report=genbank&log$=nucltop&blast_rank=3&RID=SW40K7ER01R) | 99 |
| 0.75 | 41544-4 | KC008840 | *Mortierella dichotoma* | EDFL | Zygomycetes | 863 | [JX975842](http://www.ncbi.nlm.nih.gov/nucleotide/511783619?report=genbank&log$=nucltop&blast_rank=5&RID=SWAE4FE701R) | 99 |
| 1.0 | 6719-S5 | KC008878 | *Mortierella hyalina* | EDFL | Zygomycetes | 1022 | [JX975928](http://www.ncbi.nlm.nih.gov/nucleotide/511783705?report=genbank&log$=nucltop&blast_rank=3&RID=SWAJ0WJH01R) | 100 |
| 1.0 | 38264-S4 | KC008778 | *Kernia sp.* | Ascomycota | Sordariomycetes | 604 | [DQ318208](http://www.ncbi.nlm.nih.gov/nucleotide/83779376?report=genbank&log$=nucltop&blast_rank=17&RID=SWAPMBZ801R) | 97 |
| 1.25 | *39148-S1 | KC008723 | *Cladosporium cladosporioides* | Ascomycota | Dothideomycetes | 841 | [KJ598781](http://www.ncbi.nlm.nih.gov/nucleotide/636665533?report=genbank&log$=nucltop&blast_rank=1&RID=SWAU7H6A01R) | 100 |
| 1.25 | 38258-S2 | KC008767 | [*Fusarium merismoides*](http://blast.st-va.ncbi.nlm.nih.gov/Blast.cgi#alnHdr_520688893) | Ascomycota | Sordariomycetes | 861 | [KC427027](http://www.ncbi.nlm.nih.gov/nucleotide/520688893?report=genbank&log$=nucltop&blast_rank=4&RID=SWAYYCWJ01R) | 100 |
| 1.5 | 6804-S1 | KC009075 | *Hypocrea viridescens* | Ascomycota | Sordariomycetes | 893 | [KJ482546](http://www.ncbi.nlm.nih.gov/nucleotide/625295631?report=genbank&log$=nucltop&blast_rank=2&RID=SWB8H3V301R) | 100 |
| 1.5 | 38253-S4 | KC008752 | *Mortierella amoeboidea* | EDFL | Zygomycetes | 1042 | [JX976068](http://www.ncbi.nlm.nih.gov/nucleotide/511783845?report=genbank&log$=nucltop&blast_rank=15&RID=SWBC3S5301R) | 100 |
| 1.5 | *6792-S4 | KC009014 | *Penicillium brevicompactum* | Ascomycota | Leotiomycetes | 905 | [AY373899](http://www.ncbi.nlm.nih.gov/nucleotide/34809379?report=genbank&log$=nucltop&blast_rank=2&RID=SWBH8P7301R) | 100 |
| 1.5 | *38255-S2 | KC008761 | *Mucor hiemalis* | EDFL | Zygomycetes | 1003 | \|  \| [KF944455](http://www.ncbi.nlm.nih.gov/nucleotide/576941507?report=genbank&log$=nucltop&blast_rank=2&RID=SWBNFM7201R) \| \| --- \| --- \| | 100 |
| 1.5 | 41548-1 | KC008847 | *Cadophora fastigiata* | EDFL | Zygomycetes | 889 | \|  \| [EU484297](http://www.ncbi.nlm.nih.gov/nucleotide/171467218?report=genbank&log$=nucltop&blast_rank=35&RID=SWBT1SEZ01R) \| \| --- \| --- \| | 95 |
| 1.5 | 38261-R2 | KC008775 | *Mortierella gamsii* | EDFL | Zygomycetes | 926 | \|  \| [JX975984](http://www.ncbi.nlm.nih.gov/nucleotide/511783761?report=genbank&log$=nucltop&blast_rank=12&RID=SWBWR3EZ01R) \| \| --- \| --- \| | 98 |
| 1.5 | 38259-R2 | KC008769 | *Helicostylum elegans* | EDFL | Zygomycetes | 1029 | \|  \| [AB113014](http://www.ncbi.nlm.nih.gov/nucleotide/32170781?report=genbank&log$=nucltop&blast_rank=5&RID=SWC265K001R) \| \| --- \| --- \| | 99 |
| 1.75 | 6717-S3 | KC008873 | *Phaeothecoidea sp.* | Ascomycota | Dothideomycetes | 604 | \|  \| [KC460848](http://www.ncbi.nlm.nih.gov/nucleotide/478785934?report=genbank&log$=nucltop&blast_rank=11&RID=SWC639DV01R) \| \| --- \| --- \| | 93 |
| 2.75 | *39147-R4 | KC008822 | *Helicostylum pulchrum* | EDFL | Zygomycetes | 876 | \|  \| [JN206053](http://www.ncbi.nlm.nih.gov/nucleotide/409185455?report=genbank&log$=nucltop&blast_rank=17&RID=SWCBYMUY01R) \| \| --- \| --- \| | 99 |
| 2.75 | 38273-S4 | KC008787 | [*Mortierella jenkinii*](http://blast.st-va.ncbi.nlm.nih.gov/Blast.cgi#alnHdr_511783626) | EDFL | Zygomycetes | 965 | [JX975849](http://www.ncbi.nlm.nih.gov/nucleotide/511783626?report=genbank&log$=nucltop&blast_rank=9&RID=SWCFNYPU01R) | 99 |
| 3.0 | *38250-S1 | KC008739 | *Penicillium swiecickii* | Ascomycota | Leotiomycetes | 787 | [AM236585](http://www.ncbi.nlm.nih.gov/nucleotide/118918354?report=genbank&log$=nucltop&blast_rank=7&RID=SWCNJVF701R) | 99 |
| 3.25 | 38250-S2 | KC008740 | *Mortierella sp.* | EDFL | Zygomycetes | 843 | \|  \| [JX976069](http://www.ncbi.nlm.nih.gov/nucleotide/511783846?report=genbank&log$=nucltop&blast_rank=33&RID=SWCT4XV101R) \| \| --- \| --- \| | 95 |
| 3.25 | 6785-S3 | KC008975 | *Debaryomyces maramus* | Ascomycota | Sordariomycetes | 989 | [AJ586525](http://www.ncbi.nlm.nih.gov/nucleotide/54143991?report=genbank&log$=nucltop&blast_rank=5&RID=SWCWMRHD01R) | 99 |
| 3.25 | 38254-R3 | KC008759 | *Geomyces sp.* | Ascomycota | Leotiomycetes | 658 | [NR_111872](http://www.ncbi.nlm.nih.gov/nucleotide/597901207?report=genbank&log$=nucltop&blast_rank=90&RID=SWDGZM9M01R) | 93 |
| 3.5 | *38252-R2 | KC008750 | *Oidiodendron truncatum* | Ascomycota | Leotiomycetes | 852 | \|  \| [FJ914713](http://www.ncbi.nlm.nih.gov/nucleotide/237872439?report=genbank&log$=nucltop&blast_rank=8&RID=SWDNFNP001R) \| \| --- \| --- \| | 99 |
| 7.5 | *38263-S2 | KC008776 | *Trichosporon dulcitum* | Basidiomycota | Tremellomycetes | 693 | [NR_073248](http://www.ncbi.nlm.nih.gov/nucleotide/408880877?report=genbank&log$=nucltop&blast_rank=46&RID=SWDVFF3401R) | 100 |
| 13.25 | *38251-S3 | KC008745 | *Geomyces pannorum* | Ascomycota | Leotiomycetes | 833 | [KC461530](http://www.ncbi.nlm.nih.gov/nucleotide/479327266?report=genbank&log$=nucltop&blast_rank=10&RID=SWDZ1BE601R) | 100 |
| 21 | 38249-4 | KC008731 | *Penicillium griseofulvum* | Ascomycota | Leotiomycetes | 743 | \|  \| [JX091405](http://www.ncbi.nlm.nih.gov/nucleotide/452850328?report=genbank&log$=nucltop&blast_rank=12&RID=SWE3MMBR01R) \| \| --- \| --- \| | 99 |

^a^Relative abundance for the combined libraries, which was used to sort the entries

^b^OTUs were characterized by Mothur program [1], the OTU is ≥97% similar to a fungal isolate

^c^BLASTN [2] score value

^d^Accession number of the closest database match

^e^Level of similarity for pairwise alignments with the closest match, using the Martinez-Needleman-Wunsch algorithm [2]

^*^Common OTUs identified from ITS2, ITS, and LSU sequences
