# Supplementary material for: Mycobiome of the Bat White Nose Syndrome Affected Caves and Mines Reveals Diversity of Fungi and Local Adaptation by the Fungal Pathogen Pseudogymnoascus (Geomyces) destructans
Source: PLoS One. 2014 Sep 29;9(9):e108714. doi: 10.1371/journal.pone.0108714 (PMC4181696; doi:10.1371/journal.pone.0108714)
Supplement: Table S6 — Details of LSU sequences of fungal isolates recovered by CD method. (DOCX) [file pone.0108714.s007.docx]

Table S6. Details of LSU sequences of fungal isolates recovered by CD method

| Sum  (%)^a^ | OTU^b^ | | Accession no. | Best BLAST hit  Taxon Phylum Score^c^ Acc. no.^d^ coverage | | | | | %Similiarity^e^ |
| --- | --- | --- | --- | --- | --- | --- | --- | --- | --- |
| 2.56 | | *6795-R1 | KC009247 | *Mortierella hyalina* | EDFL | 1205 | JN940867 | 100 | 99 |
| 2.56 | | 6798-S2 | KC009253 | *Penicillium roqueforti* | Ascomycota | 946 | JQ434686 | 100 | 100 |
| 2.56 | | 6799-S2 | KC009256 | *Cordyceps militaris* | Ascomycota | 428 | JQ286887 | 100 | 99 |
| 2.56 | | *6799-R3 | KC009257 | *Trichosporon dulcitum* | Basidiomycota | 992 | AF444428 | 100 | 100 |
| 2.56 | | *6801-S6 | KC009259 | *Verticillium leptobactrum* | Ascomycota | 907 | JQ410322 | 100 | 99 |
| 2.56 | | 6802-S6 | KC009260 | *Cuspidatispora xiphiago* | Ascomycota | 874 | DQ376251 | 100 | 99 |
| 2.56 | | 6803-S3 | KC009261 | *Pochonia bulbillosa* | Ascomycota | 918 | JQ780662 | 100 | 99 |
| 2.56 | | 6804-R4 | KC009262 | *Trichoderma viridescens* | Ascomycota | 880 | HM535608 | 100 | 99 |
| 2.56 | | 6805-S6 | KC009264 | *Mammaria echinobotryoides* | Ascomycota | 756 | DQ376251 | 100 | 99 |
| 2.56 | | *6805-R1 | KC009265 | *Mortierella alpina* | EDFL | 680 | AB517932 | 100 | 99 |
| 2.56 | | 6806-R3 | KC009269 | *Oidiodendron tenuissimum* | Ascomycota | 843 | AB040706 | 100 | 97 |
| 2.56 | | *6807-S2 | KC009270 | *Penicillium quercetorum* | Ascomycota | 819 | AY443471 | 100 | 99 |
| 2.56 | | *6807-S5 | KC009271 | *Kernia pachypleura* | Ascomycota | 472 | DQ318208 | 100 | 97 |
| 2.56 | | 6809-S2 | KC009275 | *Simplicillium sp.* | Ascomycota | 662 | AB378540 | 99 | 95 |
| 5.13 | | 6796-S3 | KC009248 | *Arthroderma silverae* | Ascomycota | 859 | AY176729 | 100 | 99 |
| 5.13 | | *6796-S4 | KC009249 | *Mortierella parvispora* | EDFL | 1182 | HM849689 | 100 | 99 |
| 5.13 | | 6796-R1 | KC009250 | *Thamnidium elegans* | EDFL | 1162 | AB614353 | 100 | 99 |
| 5.13 | | *6797-R1 | KC009252 | *Mortierella sarnyensis* | EDFL | 1229 | FJ161944 | 100 | 99 |
| 5.13 | | *6817-S4 | KC009284 | *Mucor hiemalis* | EDFL | 1086 | JN315041 | 100 | 99 |
| 10.26 | | 6799-R4 | KC009258 | *Coprinus callinus* | Ascomycota | 861 | AB470586 | 100 | 98 |
| 10.26 | | 6805-S3 | KC009263 | *Chrysosporium vallenarense* | Ascomycota | 904 | AY176729 | 100 | 99 |
| 17.95 | | 6808-S6 | KC009274 | *Kernia retardata* | Ascomycota | 933 | AB470603 | 98 | 99 |

^a^Relative abundance for the combined libraries, which was used to sort the entries

^b^OTUs were characterized by Mothur program [1], the OTU is ≥97% similar to a fungal isolate

^c^BLASTN [2] score value

^d^Accession number of the closest database match

^e^Level of similarity for pairwise alignments with the closest match, using the Martinez-Needleman-Wunsch algorithm [2]

^*^Common OTUs identified from ITS2, ITS, and LSU sequences
