# Supplementary material for: Mycobiome of the Bat White Nose Syndrome Affected Caves and Mines Reveals Diversity of Fungi and Local Adaptation by the Fungal Pathogen Pseudogymnoascus (Geomyces) destructans
Source: PLoS One. 2014 Sep 29;9(9):e108714. doi: 10.1371/journal.pone.0108714 (PMC4181696; doi:10.1371/journal.pone.0108714)
Supplement: Table S7 — Details of ITS sequences of fungal clones recovered by CI method. (DOCX) [file pone.0108714.s008.docx]

Table S7. Details of ITS sequences of fungal clones recovered by CI method

| Sum  (%)^a^ | OTU^b^ | | Accession no. | Best BLAST hit  Taxon Phylum Score^c^ Acc. no.^d^ Coverage ^e^ | | | | | | %Similiarity^f^ |
| --- | --- | --- | --- | --- | --- | --- | --- | --- | --- | --- |
| 0.41 | | S01-02 JX898552 | |  | Uncultured *Trichosporon* sp. clone | [Basidiomycota](http://en.wikipedia.org/wiki/Basidiomycota) | 569 | AB180201 | 74 | 99 |
| 0.41 | | S01-05 JX898607 | |  | Uncultured soil fungus clone | EDFL | 424 | JQ666464 | 47 | 92 |
| 0.41 | | S01-15 JX898609 | |  | Uncultured soil fungus clone | EDFL | 137 | EU517006 | 19 | 90 |
| 0.41 | | S01-16 JX898610 | |  | Uncultured soil fungus clone | EDFL | 292 | EF635761 | 32 | 96 |
| 0.41 | | S06-36 JX898614 | |  | Uncultured fungus clone | EDFL | 233 | EF392540 | 18 | 93 |
| 0.41 | | S06-40 JX898596 | |  | Uncultured *Hyaloraphidium* sp. clone | Chytridiomycota | 334 | AY997055 | 68 | 80 |
| 0.41 | | S07-05 JX898597 | |  | Uncultured *Sympodiomyces* sp. clone | Ascomycota | 520 | HQ623607 | 99 | 87 |
| 0.41 | | S01-17 JX898611 | |  | Uncultured [*Mortierella* sp.](http://blast.ncbi.nlm.nih.gov/Blast.cgi#alnHdr_411031012) clone | EDFL | 924 | HQ630349 | 100 | 96 |
| 0.41 | | S01-19 JX898559 | |  | Uncultured *Verrucariales* sp. clone | Ascomycota | 697 | HQ022024 | 75 | 91 |
| 0.41 | | S02-02 JX605057 | |  | Uncultured fungus clone | EDFL | 460 | FM178265 | 100 | 89 |
| 0.41 | | S01-11 JX605055 | |  | Uncultured *Trichosporon* sp. clone | Ascomycota | 767 | FJ943427 | 100 | 98 |
| 0.41 | | S01-13 JX898608 | |  | Uncultured soil fungus clone | EDFL | 169 | EU489943 | 15 | 90 |
| 0.41 | | S02-07 JX898611 | |  | Uncultured fungus clone | Ascomycota | 355 | JX974777 | 56 | 92 |
| 0.41 | | S02-16 JX605066 | |  | Uncultured *Debaryomyces sp.*clone | Ascomycota | 1046 | KC111444 | 100 | 100 |
| 0.41 | | S02-17 JX898565 | |  | Uncultured fungus clone | EDFL | 329 | FM178265 | 86 | 86 |
| 0.41 | | S02-37 JX898572 | |  | Uncultured *Trichosporon* sp. clone | [Basidiomycota](http://en.wikipedia.org/wiki/Basidiomycota) | 608 | HF558657 | 66 | 98 |
| 0.41 | | S02-38 JX898573 | |  | Uncultured [*Mortierella* sp.](http://blast.ncbi.nlm.nih.gov/Blast.cgi#alnHdr_411031012) clone | EDFL | 723 | HQ211948 | 99 | 89 |
| 0.41 | | S04-18 JX898575 | |  | Uncultured *Ganoderma* sp. clone clone | [Basidiomycota](http://en.wikipedia.org/wiki/Basidiomycota) | 654 | JN048773 | 75 | 99 |
| 0.41 | | S04-24 JX605097 | |  | Uncultured soil fungus clone | EDFL | 497 | JQ666392 | 100 | 93 |
| 0.41 | | *S04-26 JX898576 | |  | Uncultured *Penicillium sp.* clone | Ascomycota | 959 | JX270368 | 100 | 99 |
| 0.41 | | S04-34 JX605106 | |  | Uncultured soil fungus clone | EDFL | 545 | JQ666392 | 100 | 96 |
| 0.41 | | S04-40 JX898577 | |  | [Uncultured Glomeromycota clone](http://blast.ncbi.nlm.nih.gov/Blast.cgi#alnHdr_298257779) | Glomeromycota | 420 | GU392007 | 53 | 92 |
| 0.41 | | *S05-02 JX898578 | |  | Uncultured *Trichosporon dulcitum* clone | [Basidiomycota](http://en.wikipedia.org/wiki/Basidiomycota) | 815 | HM136684 | 100 | 99 |
| 0.41 | | *S05-13 JX605117 | |  | Uncultured *Thamnidium elegans* clone | EDFL | 1153 | AB113025 | 100 | 99 |
| 0.41 | | S05-16 JX605120 | |  | Uncultured *Chrysosporium* sp. clone | Ascomycota | 852 | AM949568 | 100 | 94 |
| 0.41 | | S05-18 JX898582 | |  | Uncultured Pseudeurotiaceae sp. clone | Ascomycota | 564 | JX270610 | 62 | 99 |
| 0.41 | | S05-19 JX605122 | |  | Uncultured *Geomyces* sp. clone | Ascomycota | 575 | HQ211533 | 100 | 89 |
| 0.41 | | S05-31 JX898584 | |  | Uncultured *Trichosporon* sp. clone | [Basidiomycota](http://en.wikipedia.org/wiki/Basidiomycota) | 619 | HF558657 | 66 | 99 |
| 0.41 | | S06-09 JX898590 | |  | Uncultured fungus clone | EDFL | 488 | EF635761 | 74 | 84 |
| 0.41 | | S06-11 JX605145 | |  | Uncultured Ascomycota clone | Ascomycota | 648 | HQ211979 | 92 | 96 |
| 0.41 | | S06-12 JX898591 | |  | Uncultured fungus clone | Ascomycota | 808 | EF434057 | 97 | 88 |
| 0.41 | | S06-13 JX605146 | |  | Uncultured fungus clone | EDFL | 484 | AJ920022 | 100 | 89 |
| 0.41 | | *S06-15 JX605148 | |  | Uncultured *Penicillium brevicompactum* | Ascomycota | 924 | HM469408 | 100 | 100 |
| 0.41 | | S06-16 JX898613 | |  | Uncultured soil fungus clone | Chytridiomycota | 257 | EU480336 | 26 | 94 |
| 0.41 | | S11-04 JX898619 | |  | Uncultured fungus clone | EDFL | 113 | JQ313110 | 18 | 84 |
| 0.41 | | S11-06 JX898620 | |  | Uncultured *Leptodiscella sp.clone* | EDFL | 127 | FR821312 | 17 | 83 |
| 0.41 | | S11-08 JX898621 | |  | Uncultured fungus clone | EDFL | 250 | HQ693513 | 25 | 90 |
| 0.41 | | S11-09 JX898622 | |  | Uncultured fungus clone | EDFL | 295 | EU825633 | 24 | 95 |
| 0.41 | | S11-10 JX898604 | |  | Uncultured fungus clone | EDFL | 358 | JQ681169 | 88 | 81 |
| 0.41 | | S11-12 JX898623 | |  | Uncultured fungus clone | EDFL | 134 | DQ421220 | 23 | 77 |
| 0.41 | | S11-16 JX898624 | |  | Uncultured fungus clone | EDFL | 291 | EU517006 | 28 | 97 |
| 0.41 | | S11-20 JX675217 | |  | Uncultured *Exophiala dermatitidis* clone | Ascomycota | 1059 | GU942305 | 100 | 99 |
| 0.41 | | S09-15 JX898616 | |  | Uncultured fungus clone | EDFL | 282 | HQ829352 | 26 | 90 |
| 0.41 | | S09-19 JX898603 | |  | Uncultured fungus clone | EDFL | 302 | HQ829349 | 68 | 87 |
| 0.41 | | S11-01 JX605210 | |  | Uncultured *Lycoperdon pyriforme* clone | Basidiomycota | 1157 | AY854075 | 100 | 99 |
| 0.41 | | S11-02 JX898618 | |  | Uncultured fungus clone | EDFL | 132 | EU718671 | 34 | 86 |
| 0.41 | | S07-23 JX898600 | |  | Uncultured *Mortierella* sp. Finse clone | EDFL | 733 | AJ541798 | 71 | 99 |
| 0.41 | | S09-13 JX898615 | |  | Uncultured soil fungus clone | EDFL | 197 | JQ666448 | 33 | 89 |
| 0.41 | | S06-19 JX605151 | |  | Uncultured tremellomycete clone | Basidiomycota | 994 | EU030394 | 100 | 99 |
| 0.41 | | S06-22 JX898592 | |  | Uncultured fungus clone | EDFL | 652 | EU516682 | 72 | 93 |
| 0.41 | | S09-08 JX605203 | |  | Uncultured fungus clone | Ascomycota | 668 | EU754961 | 100 | 94 |
| 0.83 | | *S11-03 JX605211 | |  | Uncultured *Ganoderma* sp. clone | Basidiomycota | 1000 | AF255097 | 100 | 99 |
| 0.83 | | S01-01 JX605050 | |  | Uncultured soil fungus clone | EDFL | 982 | GU083255 | 100 | 99 |
| 0.83 | | S01-07 JX898554 | |  | Uncultured [*Mortierella* sp.](http://blast.ncbi.nlm.nih.gov/Blast.cgi#alnHdr_411031012) clone | EDFL | 852 | JX270390 | 86 | 98 |
| 0.83 | | S01-14 JX898557 | |  | Uncultured Ascomycota clone | Ascomycota | 285 | HM240007 | 51 | 84 |
| 0.83 | | S02-18 JX605067 | |  | Uncultured [*Mortierella* *hypsicladia*](http://blast.ncbi.nlm.nih.gov/Blast.cgi#alnHdr_411031012) clone | EDFL | 1072 | HQ630302 | 100 | 98 |
| 0.83 | | S05-25 JX605127 | |  | Uncultured *Gymnoascus* sp. clone | Ascomycota | 1011 | JX270524 | 100 | 100 |
| 0.83 | | S07-09 JX605168 | |  | Uncultured *Paecilomyces inflatus* clone | Ascomycota | 868 | GU566291 | 100 | 99 |
| 0.83 | | S06-23 JX605152 | |  | Uncultured *Thamnidium elegans* clone | EDFL | 884 | AB113013 | 100 | 92 |
| 0.83 | | S04-15 JX898612 | |  | Uncultured fungus clone | EDFL | 298 | FJ528704 | 29 | 94 |
| 1.24 | | S02-03 JX898562 | |  | Uncultured  [*Gymnoascus* sp.](http://blast.ncbi.nlm.nih.gov/Blast.cgi#alnHdr_411031012) clone | Ascomycota | 1002 | JX270593 | 100 | 99 |
| 1.24 | | S09-03 JX898601 | |  | Uncultured fungus clone | EDFL | 612 | EF635761 | 89 | 93 |
| 1.24 | | *S06-17 JX605149 | |  | Uncultured *Mortierella cf. gamsii* clone | EDFL | 1023 | HQ630307 | 100 | 100 |
| 1.24 | | S06-33 JX898595 | |  | Uncultured fungus clone | EDFL | 893 | EU516682 | 92 | 92 |
| 1.66 | | *S01-06 JX605052 | |  | Uncultured [*Mortierella* sp.](http://blast.ncbi.nlm.nih.gov/Blast.cgi#alnHdr_411031012) clone | EDFL | 1020 | JX270478 | 100 | 99 |
| 1.66 | | S05-26 JX605128 | |  | Uncultured fungus clone | Chytridiomycota | 488 | EU480016 | 100 | 84 |
| 2.07 | | *S09-09 JX605204 | |  | Uncultured [*Mortierella* sp.](http://blast.ncbi.nlm.nih.gov/Blast.cgi#alnHdr_411031012) clone | EDFL | 989 | GU327518 | 100 | 99 |
| 2.07 | | *S02-21 JX605069 | |  | Uncultured *Helicostylum pulchrum* clone | EDFL | 1099 | JQ319049 | 100 | 99 |
| 2.90 | | *S05-30 JX605132 | |  | Uncultured *Chaetomium* sp. clone | Ascomycota | 881 | GU934510 | 100 | 99 |
| 2.90 | | *S06-14 JX605147 | |  | Uncultured *Geomyces pannorum* clone | Ascomycota | 895 | JF311913 | 100 | 100 |
| 8.29 | | *S02-19 JX898566 | |  | Uncultured [*Mortierella* sp.](http://blast.ncbi.nlm.nih.gov/Blast.cgi#alnHdr_411031012) clone | EDFL | 1077 | JX270382 | 100 | 99 |
| 17.84 | | *S04-33 JX605105 | |  | Uncultured *Trichosporon dulcitum* clone | [Basidiomycota](http://en.wikipedia.org/wiki/Basidiomycota) | 859 | HF558657 | 100 | 100 |
| 26.97 | | *S07-15 JX605174 | |  | Uncultured [*Mortierella* sp.](http://blast.ncbi.nlm.nih.gov/Blast.cgi#alnHdr_411031012) clone | EDFL | 1058 | AJ541798 | 100 | 99 |

^a^Relative abundance for the combined libraries, which was used to sort the entries

^b^OTUs were characterized by Mothur platform [1], the OTU is ≥97% similar to a fungal isolate

^c^BLASTN [2] score value

^d^Accession number of the closest database match

^e^Coverage of pairwise alignment of the closest database match

^f^Level of similarity for pairwise alignments with the closest match, using the Martinez-Needleman-Wunsch algorithm [2]

^*^Common OTUs recovered from LSU and ITS clone libraries
