# Supplementary material for: Mycobiome of the Bat White Nose Syndrome Affected Caves and Mines Reveals Diversity of Fungi and Local Adaptation by the Fungal Pathogen Pseudogymnoascus (Geomyces) destructans
Source: PLoS One. 2014 Sep 29;9(9):e108714. doi: 10.1371/journal.pone.0108714 (PMC4181696; doi:10.1371/journal.pone.0108714)
Supplement: Table S8 — Details of LSU sequences of fungal clones recovered by CI method. (DOCX) [file pone.0108714.s009.docx]

Table S8. Details of LSU sequences of fungal clones recovered by CI method

| Sum(%)^a^ | OTU^b^ | Accession no. | Best BLAST hit  Taxon Phylum Score^c^ Acc. no.^d^ | | | | | %Similiarity^e^ |
| --- | --- | --- | --- | --- | --- | --- | --- | --- |
| 0.53 | D06-36 | JX534740 | *Uncultured Trichosporon sp. clone* | Basidiomycota |  | 767 | [KF212338](http://www.ncbi.nlm.nih.gov/nucleotide/530692646?report=genbank&log$=nucltop&blast_rank=7&RID=SYB7667C01R) | 99 |
| 0.53 | D09-09 | JX534814 | *Uncultured Trichosporon sp. clone* | Basidiomycota |  | 669 | JN939493 | 69 |
| 0.53 | D12-35 | JX534928 | *Uncultured soil fungus clone* | EDFL |  | 1024 | \|  \| [KF56578](http://www.ncbi.nlm.nih.gov/nucleotide/583832226?report=genbank&log$=nucltop&blast_rank=13&RID=SY76YHYF015)9 \| \| --- \| --- \| | 95 |
| 0.53 | D09-31 | JX534833 | *Uncultured soil fungus clone* | Ascomycota |  | 1081 | [KF566345](http://www.ncbi.nlm.nih.gov/nucleotide/583832782?report=genbank&log$=nucltop&blast_rank=16&RID=SY8RVEX3014) | 94 |
| 0.53 | D08-30 | JX534799 | *Uncultured soil fungus clone* | EDFL |  | 1003 | \|  \| [EU516994](http://www.ncbi.nlm.nih.gov/nucleotide/187961916?report=genbank&log$=nucltop&blast_rank=20&RID=SY8VGAX8014) \| \| --- \| --- \| | 93 |
| 0.53 | D09-27 | JX534829 | *Uncultured soil fungus clone* | Ascomycota |  | 939 | [KF565913](http://www.ncbi.nlm.nih.gov/nucleotide/583832350?report=genbank&log$=nucltop&blast_rank=10&RID=SY97CAXV01R) | 87 |
| 0.53 | *D03-08 | JX534651 | *Uncultured Mortierella hyalina clone* | EDFL |  | 1173 | [JN940868](http://www.ncbi.nlm.nih.gov/nucleotide/359294441?report=genbank&log$=nucltop&blast_rank=26&RID=SYAJN4SD01R) | 98 |
| 0.53 | D08-13 | JX534785 | *Uncultured soil fungus clone* | EDFL |  | 603 | [KF566652](http://www.ncbi.nlm.nih.gov/nucleotide/583833089?report=genbank&log$=nucltop&blast_rank=3&RID=SYAPGJY101R) | 99 |
| 0.53 | D03-23 | JX534664 | *Uncultured M.* [*exigua voucher*](http://blast.st-va.ncbi.nlm.nih.gov/Blast.cgi#alnHdr_223929953) *clone* | EDFL |  | 1243 | \|  \| [FJ161943](http://www.ncbi.nlm.nih.gov/nucleotide/223929953?report=genbank&log$=nucltop&blast_rank=5&RID=SYAVNU1W01R) \| \| --- \| --- \| | 99 |
| 0.53 | D03-40 | JX534677 | [*Uncultured Trichosporon clon*](http://blast.st-va.ncbi.nlm.nih.gov/Blast.cgi#alnHdr_408354674)*e* | Basidiomycota |  | 798 | [JN939493](http://www.ncbi.nlm.nih.gov/nucleotide/358441577?report=genbank&log$=nucltop&blast_rank=8&RID=SYB1PWEA01R) | 93 |
| 0.53 | D09-15 | JX534820 | *Uncultured soil fungus clone* | EDFL |  | 575 | [KF565528](http://www.ncbi.nlm.nih.gov/nucleotide/583831965?report=genbank&log$=nucltop&blast_rank=10&RID=SYBDHSU901R) | 66 |
| 0.53 | D04-10 | JX534682 | *Uncultured Trichosporon sp. clone* | Basidiomycota |  | 630 | [JN939493](http://www.ncbi.nlm.nih.gov/nucleotide/358441577?report=genbank&log$=nucltop&blast_rank=23&RID=SYBHSTJ901R) | 93 |
| 0.53 | D07-06 | JX534747 | *Uncultured Guehomyces pullulans clone* | Basidiomycota |  | 981 | [GQ202976](http://www.ncbi.nlm.nih.gov/nucleotide/240013010?report=genbank&log$=nucltop&blast_rank=5&RID=SYBN0PAE01R) | 100 |
| 0.53 | D10-02 | JX534840 | *Uncultured Hypochniciellum molle clone* | Basidiomycota |  | 992 | [GU187667](http://www.ncbi.nlm.nih.gov/nucleotide/300394471?report=genbank&log$=nucltop&blast_rank=8&RID=SYBSKMW601R) | 99 |
| 0.53 | D01-07 | JX534607 | *Uncultured Mortierella sp.clone* | EDFL |  | 1009 | [JX976166](http://www.ncbi.nlm.nih.gov/nucleotide/511783943?report=genbank&log$=nucltop&blast_rank=5&RID=SYBVVKK901R) | 94 |
| 0.53 | D01-34 | JX534625 | *Uncultured Mortierella sp.clone* | EDFL |  | 780 | [HQ667424](http://www.ncbi.nlm.nih.gov/nucleotide/357089532?report=genbank&log$=nucltop&blast_rank=16&RID=SYBYNWGU01R) | 72 |
| 0.53 | D03-03 | JX534647 | *Uncultured Trichosporon sp. clone* | Basidiomycota |  | 579 | [JN939493](http://www.ncbi.nlm.nih.gov/nucleotide/358441577?report=genbank&log$=nucltop&blast_rank=27&RID=SYC3676101R) | 66 |
| 0.53 | D01-04 | JX534605 | *Uncultured Doratomyces stemonitis clone* | Ascomycota |  | 933 | [DQ836907](http://www.ncbi.nlm.nih.gov/nucleotide/110810455?report=genbank&log$=nucltop&blast_rank=3&RID=SYC73X2K01R) | 99 |
| 0.53 | D09-06 | JX534812 | *Uncultured soil fungus clone* | EDFL |  | 881 | [KF566043](http://www.ncbi.nlm.nih.gov/nucleotide/583832480?report=genbank&log$=nucltop&blast_rank=4&RID=SYCAYHFG01R) | 90 |
| 0.53 | D06-25 | JX534735 | *Uncultured* [*Basidiomycota*](http://blast.st-va.ncbi.nlm.nih.gov/Blast.cgi#alnHdr_85002494) *clone* | Basidiomycota |  | 841 | \|  \| [DQ341804](http://www.ncbi.nlm.nih.gov/nucleotide/85002494?report=genbank&log$=nucltop&blast_rank=2&RID=SYCDXBUG01R) \| \| --- \| --- \| | 95 |
| 0.53 | D03-26 | JX534666 | *Uncultured Mortierella sp. clone* | EDFL |  | 1122 | [JN940866](http://www.ncbi.nlm.nih.gov/nucleotide/359294439?report=genbank&log$=nucltop&blast_rank=10&RID=SYCHNHHS01R) | 96 |
| 0.53 | D03-01 | JX534646 | *Uncultured Mortierella sp. clone* | EDFL |  | 915 | [KC018415](http://www.ncbi.nlm.nih.gov/nucleotide/511784186?report=genbank&log$=nucltop&blast_rank=21&RID=SYCNSETZ01R) | 92 |
| 0.53 | D03-06 | JX534649 | *Uncultured Mortierella sp. clone* | EDFL |  | 1081 | [KC018408](http://www.ncbi.nlm.nih.gov/nucleotide/511784179?report=genbank&log$=nucltop&blast_rank=22&RID=SYCSNPW501R) | 96 |
| 0.53 | D05-24 | JX534711 | *Uncultured Mortierella sp. clone* | EDFL |  | 822 | \|  \| [FJ161938](http://www.ncbi.nlm.nih.gov/nucleotide/223929948?report=genbank&log$=nucltop&blast_rank=17&RID=SYCVJN2R01R) \| \| --- \| --- \| | 88 |
| 0.53 | D11-25 | JX534895 | *Uncultured Coprinellus micaceus clone* | Basidiomycota |  | 1005 | [AY207182](http://www.ncbi.nlm.nih.gov/nucleotide/62946856?report=genbank&log$=nucltop&blast_rank=4&RID=SYE4VRKW01R) | 99 |
| 0.53 | *D05-04 | JX534701 | *Uncultured* [*Mortierella indohii*](http://blast.st-va.ncbi.nlm.nih.gov/Blast.cgi#alnHdr_209402438) *clone* | EDFL |  | 1149 | [EU736318](http://www.ncbi.nlm.nih.gov/nucleotide/209402438?report=genbank&log$=nucltop&blast_rank=17&RID=SYED6Y3D01R) | 98 |
| 0.53 | D11-21 | JX534892 | *Uncultured Mortierella polycephala clone* | EDFL |  | 1192 | [KC018297](http://www.ncbi.nlm.nih.gov/nucleotide/511784068?report=genbank&log$=nucltop&blast_rank=15&RID=SYEJ03TD01R) | 97 |
| 0.53 | D09-38 | JX534837 | *Uncultured Trichosporon sp. clone* | Basidiomycota |  | 599 | [JN939493](http://www.ncbi.nlm.nih.gov/nucleotide/358441577?report=genbank&log$=nucltop&blast_rank=23&RID=SYENT3XE01R) | 65 |
| 0.53 | D05-16 | JX534707 | *Uncultured Trichosporon middelhovenii clone* | Basidiomycota |  | 937 | [AB180198](http://www.ncbi.nlm.nih.gov/nucleotide/47776291?report=genbank&log$=nucltop&blast_rank=2&RID=SYESDJGT01R) | 99 |
| 0.53 | D09-29 | JX534831 | *Uncultured Fusarium sp. clone* | Ascomycota |  | 486 | \|  \| [EU860057](http://www.ncbi.nlm.nih.gov/nucleotide/218455080?report=genbank&log$=nucltop&blast_rank=12&RID=SYEW32G101R) \| \| --- \| --- \| | 62 |
| 0.53 | *D03-33 | JX534672 | *Uncultured Mortierella gamsii clone* | EDFL |  | 1236 | \|  \| [HQ667384](http://www.ncbi.nlm.nih.gov/nucleotide/357089492?report=genbank&log$=nucltop&blast_rank=3&RID=SYEZEPGD01R) \| \| --- \| --- \| | 100 |
| 0.53 | D10-16 | JX534853 | *Uncultured Mortierella polycephala clone* | EDFL |  | 1212 | [JN939145](http://www.ncbi.nlm.nih.gov/nucleotide/358441414?report=genbank&log$=nucltop&blast_rank=2&RID=SYF343TC01R) | 99 |
| 0.53 | D01-21 | JX534617 | *Uncultured soil fungus clone* | EDFL |  | 612 | [KC965494](http://www.ncbi.nlm.nih.gov/nucleotide/532165651?report=genbank&log$=nucltop&blast_rank=3&RID=SYF69UMV01R) | 88 |
| 0.53 | D05-40 | JX534724 | *Uncultured soil fungus clone* | Ascomycota |  | 529 | [KJ150290](http://www.ncbi.nlm.nih.gov/nucleotide/605052146?report=genbank&log$=nucltop&blast_rank=4&RID=SYFA0SDB01R) | 82 |
| 0.53 | D01-03 | JX534604 | *Uncultured* [*Gyromitus sp. clone*](http://blast.st-va.ncbi.nlm.nih.gov/Blast.cgi#alnHdr_407196929) | Ascomycota |  | 411 | \|  \| \|  \| [EF681955](http://www.ncbi.nlm.nih.gov/nucleotide/161015721?report=genbank&log$=nucltop&blast_rank=13&RID=SYFDE7HV01R) \| \| --- \| --- \| \| \| --- \| --- \| --- \| --- \| | 68 |
| 0.53 | D12-38 | JX534930 | *Uncultured Pluteus sp. clone* | Basidiomycota |  | 634 | \|  \| [HM562242](http://www.ncbi.nlm.nih.gov/nucleotide/308745314?report=genbank&log$=nucltop&blast_rank=3&RID=SYFKEMGJ01R) \| \| --- \| --- \| | 89 |
| 0.53 | D12-36 | JX534929 | *Uncultured soil fungus clone* | Ascomycota |  | 806 | \|  \| [JX043247](http://www.ncbi.nlm.nih.gov/nucleotide/396581025?report=genbank&log$=nucltop&blast_rank=7&RID=SYFP7BJT01R) \| \| --- \| --- \| | 88 |
| 0.53 | D08-35 | JX534804 | *Uncultured soil fungus clone* | EDFL |  | 599 | [EU489955](http://www.ncbi.nlm.nih.gov/nucleotide/170516759?report=genbank&log$=nucltop&blast_rank=20&RID=SYFUB4GV01R) | 94 |
| 0.53 | D06-01 | JX534725 | *Uncultured soil fungus clone* | EDFL |  | 656 | \|  \| [EU489955](http://www.ncbi.nlm.nih.gov/nucleotide/170516759?report=genbank&log$=nucltop&blast_rank=20&RID=SYFYT92701R) \| \| --- \| --- \| | 93 |
| 0.53 | D11-15 | JX534888 | *Uncultured soil fungus clone* | EDFL |  | 475 | \|  \| [GU376391](http://www.ncbi.nlm.nih.gov/nucleotide/289431745?report=genbank&log$=nucltop&blast_rank=12&RID=SYHWKGNH01R) \| \| --- \| --- \| | 70 |
| 0.53 | D05-30 | JX534717 | *Uncultured Gymnascella sp. clone* | Ascomycota |  | 566 | \|  \| [KC009164](http://www.ncbi.nlm.nih.gov/nucleotide/530253039?report=genbank&log$=nucltop&blast_rank=4&RID=SYMRVJTB01R) \| \| --- \| --- \| | 64 |
| 0.53 | D08-16 | JX534788 | *Uncultured soil fungus clone* | EDFL |  | 662 | \|  \| [DQ901000](http://www.ncbi.nlm.nih.gov/nucleotide/116272342?report=genbank&log$=nucltop&blast_rank=2&RID=SYMVG6Y101R) \| \| --- \| --- \| | 69 |
| 0.53 | D11-33 | JX534899 | *Uncultured soil fungus clone* | EDFL |  | 590 | [JQ311729](http://www.ncbi.nlm.nih.gov/nucleotide/375156380?report=genbank&log$=nucltop&blast_rank=3&RID=SYMYVHNA01R) | 88 |
| 0.53 | D02-07 | JX534632 | *Uncultured Geomyces destructans clone* | Ascomycota |  | 922 | \|  \| [KF212357](http://www.ncbi.nlm.nih.gov/nucleotide/530692665?report=genbank&log$=nucltop&blast_rank=4&RID=SYN233S501R) \| \| --- \| --- \| | 99 |
| 0.53 | D09-22 | JX534822 | *Uncultured Maunachytrium sp. clone* | Chytridiomycota |  | 630 | \|  \| [EF432822](http://www.ncbi.nlm.nih.gov/nucleotide/257412776?report=genbank&log$=nucltop&blast_rank=7&RID=SYN5ZPWN01R) \| \| --- \| --- \| | 86 |
| 0.53 | D09-05 | JX534811 | *Uncultured* [*Chytridiales sp.*](http://blast.st-va.ncbi.nlm.nih.gov/Blast.cgi#alnHdr_257412776) *clone* | Chytridiomycota |  | 472 | \|  \| \|  \| [EF443139](http://www.ncbi.nlm.nih.gov/nucleotide/148829213?report=genbank&log$=nucltop&blast_rank=17&RID=SYN9JSXF01R) \| \| --- \| --- \| \| \| --- \| --- \| --- \| --- \| | 81 |
| 0.53 | D12-26 | JX534922 | *Uncultured Saccharomyces cerevisiae clone* | Ascomycota |  | 961 | [KJ506733](http://www.ncbi.nlm.nih.gov/nucleotide/632799651?report=genbank&log$=nucltop&blast_rank=2&RID=SYNF9C4M01R) | 99 |
| 0.53 | D11-04 | JX534880 | *Uncultured Arthrographis sp. clone* | Ascomycota |  | 741 | [AB116543](http://www.ncbi.nlm.nih.gov/nucleotide/111378236?report=genbank&log$=nucltop&blast_rank=3&RID=SYNJ1V1K01R) | 93 |
| 0.53 | D10-19 | JX534856 | *Uncultured Acremonium sp. clone* | Ascomycota |  | 549 | \|  \| [KJ194116](http://www.ncbi.nlm.nih.gov/nucleotide/608072418?report=genbank&log$=nucltop&blast_rank=4&RID=SYNP55WH01R) \| \| --- \| --- \| | 96 |
| 0.53 | D11-34 | JX534900 | *Uncultured soil fungus clone* | EDFL |  | 470 | \|  \| [JX898660](http://www.ncbi.nlm.nih.gov/nucleotide/511488264?report=genbank&log$=nucltop&blast_rank=26&RID=SYNT6FBP01R) \| \| --- \| --- \| | 89 |
| 0.53 | D01-14 | JX534611 | *Uncultured soil fungus clone* | EDFL |  | 627 | \|  \| [DQ900984](http://www.ncbi.nlm.nih.gov/nucleotide/116272326?report=genbank&log$=nucltop&blast_rank=3&RID=SYNX98N301R) \| \| --- \| --- \| | 80 |
| 0.53 | D02-01 | JX534627 | *Uncultured soil fungus clone* | EDFL |  | 928 | \|  \| [JQ310857](http://www.ncbi.nlm.nih.gov/nucleotide/375155508?report=genbank&log$=nucltop&blast_rank=3&RID=SYPC9BHN01R) \| \| --- \| --- \| | 95 |
| 0.53 | *D02-03 | JX534628 | *Uncultured Mortierella indohii clone* | EDFL |  | 1315 | \|  \| [EU688966](http://www.ncbi.nlm.nih.gov/nucleotide/189031489?report=genbank&log$=nucltop&blast_rank=2&RID=SYPE8NKR01R) \| \| --- \| --- \| | 99 |
| 0.53 | D06-27 | JX534737 | *Uncultured soil fungus clone* | Ascomycota |  | 1162 | [KF566486](http://www.ncbi.nlm.nih.gov/nucleotide/583832923?report=genbank&log$=nucltop&blast_rank=7&RID=SYPHHADA01R) | 97 |
| 0.53 | D06-30 | JX534739 | *Uncultured soil fungus clone* | Ascomycota |  | 652 | [JQ311054](http://www.ncbi.nlm.nih.gov/nucleotide/375155705?report=genbank&log$=nucltop&blast_rank=5&RID=SYPM2WUK01R) | 75 |
| 0.53 | *D10-01 | JX534839 | *Uncultured Mortierella indohii clone* | EDFL |  | 1186 | [KC018415](http://www.ncbi.nlm.nih.gov/nucleotide/511784186?report=genbank&log$=nucltop&blast_rank=15&RID=SYPWWSB201R) | 99 |
| 0.53 | D09-28 | JX534830 | *Uncultured Zygoascus sp. clone* | Ascomycota |  | 693 | [HM036077](http://www.ncbi.nlm.nih.gov/nucleotide/296248921?report=genbank&log$=nucltop&blast_rank=12&RID=SYR241JH01R) | 88 |
| 0.53 | D02-37 | JX534643 | *Uncultured soil fungus clone* | EDFL |  | 977 | [KC965607](http://www.ncbi.nlm.nih.gov/nucleotide/532165764?report=genbank&log$=nucltop&blast_rank=2&RID=SYR6556K01R) | 96 |
| 0.53 | D08-24 | JX534795 | *Uncultured soil fungus clone* | EDFL |  | 939 | [JQ311523](http://www.ncbi.nlm.nih.gov/nucleotide/375156174?report=genbank&log$=nucltop&blast_rank=3&RID=SYRWFG5H01R) | 85 |
| 0.53 | D02-38 | JX534644 | *Uncultured soil fungus clone* | EDFL |  | 512 | \|  \| [JX534845](http://www.ncbi.nlm.nih.gov/nucleotide/408354852?report=genbank&log$=nucltop&blast_rank=4&RID=SYRZ5R7601R) \| \| --- \| --- \| | 65 |
| 0.53 | D09-23 | JX534825 | *Uncultured Pichia sp. clone* | Ascomycota |  | 787 | \|  \| [HM036077](http://www.ncbi.nlm.nih.gov/nucleotide/296248921?report=genbank&log$=nucltop&blast_rank=13&RID=SYWFT94Z01R) \| \| --- \| --- \| | 92 |
| 0.53 | D05-31 | JX534718 | *Uncultured Torrubiella wallacei clone* | Ascomycota |  | 856 | [AY184967](http://www.ncbi.nlm.nih.gov/nucleotide/37196537?report=genbank&log$=nucltop&blast_rank=3&RID=SYWXSUPC01R) | 97 |
| 0.53 | D02-05 | JX534630 | *Uncultured soil fungus clone* | EDFL |  | 442 | [KF565861](http://www.ncbi.nlm.nih.gov/nucleotide/583832298?report=genbank&log$=nucltop&blast_rank=24&RID=SYS298Z401R) | 64 |
| 0.53 | D01-29 | JX534621 | *Uncultured soil fungus clone* | EDFL |  | 590 | \|  \| JX545259 \| \| --- \| --- \| | 88 |
| 0.53 | *D10-24 | JX534860 | *Uncultured Penicillium sp. clone* | Ascomycota |  | 939 | [KF880926](http://www.ncbi.nlm.nih.gov/nucleotide/584299186?report=genbank&log$=nucltop&blast_rank=2&RID=SYX1R3JV01R) | 99 |
| 0.53 | D10-25 | JX534861 | *Uncultured Neogymnomyces sp.clone* | Ascomycota |  | 867 | [AY176716](http://www.ncbi.nlm.nih.gov/nucleotide/33113357?report=genbank&log$=nucltop&blast_rank=2&RID=SYX65D8S01R) | 97 |
| 0.53 | D10-30 | JX534866 | *Uncultured Acremonium charticola clone* | Ascomycota |  | 918 | \|  \| [HQ232015](http://www.ncbi.nlm.nih.gov/nucleotide/336180901?report=genbank&log$=nucltop&blast_rank=2&RID=SYX88ZH401R) \| \| --- \| --- \| | 99 |
| 0.53 | D10-38 | JX534874 | *Uncultured Chrysosporium sulfureum clone* | Ascomycota |  | 950 | [KC989731](http://www.ncbi.nlm.nih.gov/nucleotide/563257370?report=genbank&log$=nucltop&blast_rank=2&RID=SYXBFYSC01R) | 99 |
| 0.53 | D09-02 | JX534809 | *Uncultured soil fungus clone* | EDFL |  | 992 | \|  \| [EU861720](http://www.ncbi.nlm.nih.gov/nucleotide/194475313?report=genbank&log$=nucltop&blast_rank=6&RID=SYVY7RW101R) \| \| --- \| --- \| | 90 |
| 0.53 | D04-27 | JX534690 | *Uncultured soil fungus clone* | EDFL |  | 658 | [EU861720](http://www.ncbi.nlm.nih.gov/nucleotide/194475313?report=genbank&log$=nucltop&blast_rank=5&RID=SYW6JJCF01R) | 82 |
| 0.53 | D06-29 | JX534738 | *Uncultured soil fungus clone* | EDFL |  | 833 | [JQ311621](http://www.ncbi.nlm.nih.gov/nucleotide/375156272?report=genbank&log$=nucltop&blast_rank=2&RID=SYWATY2D01R) | 82 |
| 0.53 | D10-39 | JX534875 | *Uncultured Doratomyces sp. clone* | Ascomycota |  | 675 | \|  \| [DQ836907](http://www.ncbi.nlm.nih.gov/nucleotide/110810455?report=genbank&log$=nucltop&blast_rank=35&RID=SYXCWVGT01R) \| \| --- \| --- \| | 90 |
| 0.53 | D08-37 | JX534805 | *Uncultured Fusarium sp. clone* | Ascomycota |  | 830 | \|  \| [EU860057](http://www.ncbi.nlm.nih.gov/nucleotide/218455080?report=genbank&log$=nucltop&blast_rank=5&RID=SYXH1FAF01R) \| \| --- \| --- \| | 95 |
| 0.53 | *D07-37 | JX534773 | *Uncultured* [*Geomyces pannorum*](http://blast.st-va.ncbi.nlm.nih.gov/Blast.cgi#alnHdr_294959350) *clone* | Ascomycota |  | 909 | [GU951697](http://www.ncbi.nlm.nih.gov/nucleotide/294959354?report=genbank&log$=nucltop&blast_rank=23&RID=SYXPNZGG01R) | 99 |
| 0.53 | D08-05 | JX534779 | *Uncultured Geomyces destructans clone* | Ascomycota |  | 911 | \|  \| [KC171321](http://www.ncbi.nlm.nih.gov/nucleotide/441037390?report=genbank&log$=nucltop&blast_rank=5&RID=SYXUKW9801R) \| \| --- \| --- \| | 98 |
| 0.53 | D05-22 | JX534709 | *Uncultured* [*Chrysosporium merdarium*](http://blast.st-va.ncbi.nlm.nih.gov/Blast.cgi#alnHdr_563257367) *clone* | Ascomycota |  | 665 | \|  \| [KC989728](http://www.ncbi.nlm.nih.gov/nucleotide/563257367?report=genbank&log$=nucltop&blast_rank=6&RID=SYXZ8W5501R) \| \| --- \| --- \| | 90 |
| 0.53 | D05-27 | JX534714 | *Uncultured Amauroascus sp. clone* | Ascomycota |  | 865 | [AB075324](http://www.ncbi.nlm.nih.gov/nucleotide/32127718?report=genbank&log$=nucltop&blast_rank=3&RID=SYY4KW8901R) | 97 |
| 0.53 | D05-01 | JX534698 | *Uncultured Geomyces pannorum clone* | Ascomycota |  | 835 | [GU951694](http://www.ncbi.nlm.nih.gov/nucleotide/294959351?report=genbank&log$=nucltop&blast_rank=2&RID=SYY8AHAN01R) | 97 |
| 0.53 | D03-32 | JX534671 | *Uncultured Cercophora sparsa clone* | Ascomycota |  | 856 | [AY587937](http://www.ncbi.nlm.nih.gov/nucleotide/51104867?report=genbank&log$=nucltop&blast_rank=4&RID=SYYB88S601R) | 97 |
| 0.53 | D07-22 | JX534761 | *Uncultured Oidiodendron sp. clone* | Ascomycota |  | 893 | [AB040706](http://www.ncbi.nlm.nih.gov/nucleotide/17221589?report=genbank&log$=nucltop&blast_rank=9&RID=SYYEGSXT01R) | 98 |
| 0.53 | D01-01 | JX534602 | *Uncultured Mortierella sp. clone* | EDFL |  | 773 | [KC018389](http://www.ncbi.nlm.nih.gov/nucleotide/511784160?report=genbank&log$=nucltop&blast_rank=2&RID=SYYJSUJ301R) | 73 |
| 0.53 | D02-16 | JX534635 | *Uncultured Leuconeurospora sp. clone* | Ascomycota |  | 839 | \|  \| [AF096193](http://www.ncbi.nlm.nih.gov/nucleotide/5006397?report=genbank&log$=nucltop&blast_rank=7&RID=SYYP60BF01R) \| \| --- \| --- \| | 96 |
| 0.53 | D10-20 | JX534857 | *Uncultured Oidiodendron sp. clone* | Ascomycota |  | 736 | \|  \| [AB040706](http://www.ncbi.nlm.nih.gov/nucleotide/17221589?report=genbank&log$=nucltop&blast_rank=3&RID=SYYT90Y901R) \| \| --- \| --- \| | 93 |
| 0.53 | D10-31 | JX534867 | *Uncultured Acremonium sp. clone* | Ascomycota |  | 806 | \|  \| [HQ231987](http://www.ncbi.nlm.nih.gov/nucleotide/336180873?report=genbank&log$=nucltop&blast_rank=2&RID=SYYXZ7MH01R) \| \| --- \| --- \| | 96 |
| 0.53 | D11-17 | JX534889 | *Uncultured* [*Calycellina populina*](http://blast.st-va.ncbi.nlm.nih.gov/Blast.cgi#alnHdr_345540032) *clone* | Ascomycota |  | 928 | \|  \| [JN086693](http://www.ncbi.nlm.nih.gov/nucleotide/345540040?report=genbank&log$=nucltop&blast_rank=3&RID=SYZ1TJG601R) \| \| --- \| --- \| | 98 |
| 0.53 | D07-11 | JX534751 | [*Uncultured Cephalotheca sp. clone*](http://blast.st-va.ncbi.nlm.nih.gov/Blast.cgi#alnHdr_408354775) | Ascomycota |  | 616 | [FJ808681](http://www.ncbi.nlm.nih.gov/nucleotide/258640202?report=genbank&log$=nucltop&blast_rank=7&RID=SYZ7E04601R) | 66 |
| 0.53 | D07-20 | JX534759 | *Uncultured* [*Cephalotheca sulfurea*](http://blast.st-va.ncbi.nlm.nih.gov/Blast.cgi#alnHdr_258640202) *clone* | Ascomycota |  | 852 | [FJ808681](http://www.ncbi.nlm.nih.gov/nucleotide/258640202?report=genbank&log$=nucltop&blast_rank=5&RID=SYZB9AY801R) | 97 |
| 0.53 | D07-17 | JX534756 | *Uncultured* [*Cephalotheca sulfurea*](http://blast.st-va.ncbi.nlm.nih.gov/Blast.cgi#alnHdr_258640202) *clone* | Ascomycota |  | 883 | \|  \| [KC311468](http://www.ncbi.nlm.nih.gov/nucleotide/459354688?report=genbank&log$=nucltop&blast_rank=6&RID=SYZEP6JJ01R) \| \| --- \| --- \| | 98 |
| 0.53 | D07-35 | JX534772 | [*Uncultured Cephalotheca sp. clone*](http://blast.st-va.ncbi.nlm.nih.gov/Blast.cgi#alnHdr_408354775) | Ascomycota |  | 885 | [FJ808681](http://www.ncbi.nlm.nih.gov/nucleotide/258640202?report=genbank&log$=nucltop&blast_rank=4&RID=SYZH9Z8001R) | 98 |
| 0.53 | D08-38 | JX534806 | *Uncultured Doratomyces sp. clone* | Ascomycota |  | 876 | \|  \| [KC009280](http://www.ncbi.nlm.nih.gov/nucleotide/530253155?report=genbank&log$=nucltop&blast_rank=5&RID=SYZT6WU601R) \| \| --- \| --- \| | 97 |
| 0.53 | D09-11 | JX534816 | *Uncultured Chalara sp. clone* | Ascomycota |  | 625 | \|  \| [FJ176257](http://www.ncbi.nlm.nih.gov/nucleotide/224466352?report=genbank&log$=nucltop&blast_rank=5&RID=SYZW76UY01R) \| \| --- \| --- \| | 88 |
| 0.53 | D10-37 | JX534873 | *Uncultured Doratomyces sp. clone* | Ascomycota |  | 863 | [AB470586](http://www.ncbi.nlm.nih.gov/nucleotide/227215113?report=genbank&log$=nucltop&blast_rank=9&RID=SYZZGH9901R) | 98 |
| 0.53 | D08-34 | JX534803 | *Uncultured Chalara sp. clone* | Ascomycota |  | 774 | [FJ176257](http://www.ncbi.nlm.nih.gov/nucleotide/224466352?report=genbank&log$=nucltop&blast_rank=3&RID=SZ02S72Z01R) | 93 |
| 0.53 | D06-09 | JX534729 | *Uncultured Anisomeridium sp. clone* | Ascomycota |  | 791 | \|  \| [DQ782906](http://www.ncbi.nlm.nih.gov/nucleotide/110666891?report=genbank&log$=nucltop&blast_rank=3&RID=SZ05V9W701R) \| \| --- \| --- \| | 95 |
| 0.53 | D03-38 | JX534675 | *Uncultured Arthrobotrys superba clone* | Ascomycota |  | 931 | [EF445988](http://www.ncbi.nlm.nih.gov/nucleotide/134147771?report=genbank&log$=nucltop&blast_rank=4&RID=SZ0B2EMV01R) | 99 |
| 0.53 | D02-39 | JX534645 | *Uncultured Chalara sp. clone* | Ascomycota |  | 802 | \|  \| [FJ176257](http://www.ncbi.nlm.nih.gov/nucleotide/224466352?report=genbank&log$=nucltop&blast_rank=4&RID=SZ0ERR1R01R) \| \| --- \| --- \| | 94 |
| 0.53 | D02-20 | JX534637 | *Uncultured Chalara sp. clone* | Ascomycota |  | 689 | \|  \| [FJ176257](http://www.ncbi.nlm.nih.gov/nucleotide/224466352?report=genbank&log$=nucltop&blast_rank=4&RID=SZ0J7WXB01R) \| \| --- \| --- \| | 91 |
| 0.53 | D09-07 | JX534813 | *Uncultured Kotlabaea sp. clone* | Ascomycota |  | 926 | \|  \| [DQ220356](http://www.ncbi.nlm.nih.gov/nucleotide/81302619?report=genbank&log$=nucltop&blast_rank=5&RID=SZ0UDAZE014) \| \| --- \| --- \| | 99 |
| 0.53 | D11-23 | JX534894 | *Uncultured Cosmospora viliuscula clone* | Ascomycota |  | 935 | \|  \| [KC291785](http://www.ncbi.nlm.nih.gov/nucleotide/453756721?report=genbank&log$=nucltop&blast_rank=2&RID=SZ0Y4ZMY01R) \| \| --- \| --- \| | 99 |
| 0.53 | D12-03 | JX534907 | *Uncultured* [*Acremonium aff. curvulum*](http://blast.st-va.ncbi.nlm.nih.gov/Blast.cgi#alnHdr_336180917) *clone* | Ascomycota |  | 931 | [HQ232031](http://www.ncbi.nlm.nih.gov/nucleotide/336180917?report=genbank&log$=nucltop&blast_rank=4&RID=SZ1269BM01R) | 99 |
| 0.53 | D04-05 | JX534680 | *Uncultured Kotlabaea sp. clone* | Ascomycota |  | 922 | [DQ220356](http://www.ncbi.nlm.nih.gov/nucleotide/81302619?report=genbank&log$=nucltop&blast_rank=5&RID=SZ16BJRS01R) | 98 |
| 0.53 | D03-19 | JX534661 | *Uncultured Heydenia alpina clone* | Ascomycota |  | 907 | [HQ596526](http://www.ncbi.nlm.nih.gov/nucleotide/313510059?report=genbank&log$=nucltop&blast_rank=2&RID=SZ19VE6K01R) | 100 |
| 0.53 | D07-32 | JX898649 | *Uncultured Cephalotheca sp. clone* | Ascomycota |  | 555 | \|  \| [KC311468](http://www.ncbi.nlm.nih.gov/nucleotide/459354688?report=genbank&log$=nucltop&blast_rank=27&RID=SZ418BMS01R) \| \| --- \| --- \| | 65 |
| 0.53 | D09-03 | JX898653 | *Uncultured soil fungus clone* | Glomeromycota |  | 375 | \|  \| [KF566643](http://www.ncbi.nlm.nih.gov/nucleotide/583833080?report=genbank&log$=nucltop&blast_rank=14&RID=SZ47150R01R) \| \| --- \| --- \| | 55 |
| 0.53 | D11-14 | JX898660 | *Uncultured soil fungus clone* | Ascomycota |  | 414 | [JX067943](http://www.ncbi.nlm.nih.gov/nucleotide/398802996?report=genbank&log$=nucltop&blast_rank=30&RID=SZ4CF8FH01R) | 70 |
| 0.53 | D11-30 | JX898664 | *Uncultured soil fungus clone* | EDFL |  | 279 | [KF651080](http://www.ncbi.nlm.nih.gov/nucleotide/567569737?report=genbank&log$=nucltop&blast_rank=12&RID=SZ4HC7RC01R) | 61 |
| 0.53 | D09-18 | JX898654 | *Uncultured soil fungus clone* | EDFL |  | 436 | [KF568205](http://www.ncbi.nlm.nih.gov/nucleotide/583834639?report=genbank&log$=nucltop&blast_rank=2&RID=SZ4PD7WB01R) | 89 |
| 0.53 | D06-14 | JX898644 | *Uncultured soil fungus clone* | Glomeromycota |  | 217 | \|  \| [JN049542](http://www.ncbi.nlm.nih.gov/nucleotide/354683735?report=genbank&log$=nucltop&blast_rank=18&RID=SZ4UGVKT01R) \| \| --- \| --- \| | 91 |
| 0.53 | D06-24 | JX898645 | *Uncultured Trichosporon sp. clone* | Basidiomycota |  | 616 | [JN939493](http://www.ncbi.nlm.nih.gov/nucleotide/358441577?report=genbank&log$=nucltop&blast_rank=6&RID=SZ4XN78N01R) | 66 |
| 0.53 | D09-37 | JX898656 | *Uncultured soil fungus clone* | Chytridiomycota |  | 285 | [EU379177](http://www.ncbi.nlm.nih.gov/nucleotide/166178807?report=genbank&log$=nucltop&blast_rank=6&RID=SZ51RH1201R) | 91 |
| 0.53 | D08-26 | JX898651 | *Uncultured soil fungus clone* | EDFL |  | 392 | [KF568432](http://www.ncbi.nlm.nih.gov/nucleotide/583834866?report=genbank&log$=nucltop&blast_rank=5&RID=SZ568K7M01R) | 87 |
| 0.53 | D08-27 | JX898652 | *Uncultured Microascus sp. clone* | Ascomycota |  | 510 | [KC009283](http://www.ncbi.nlm.nih.gov/nucleotide/530253158?report=genbank&log$=nucltop&blast_rank=4&RID=SZ5B2R8Y01R) | 94 |
| 0.53 | D08-03 | JX898650 | *Uncultured soil fungus clone* | Ascomycota |  | 257 | [GU928603](http://www.ncbi.nlm.nih.gov/nucleotide/291263501?report=genbank&log$=nucltop&blast_rank=3&RID=SZ5D7JD601R) | 80 |
| 0.53 | D09-19 | JX898655 | *Uncultured soil fungus clone* | Ascomycota |  | 180 | [KF738159](http://www.ncbi.nlm.nih.gov/nucleotide/572921232?report=genbank&log$=nucltop&blast_rank=3&RID=SZ5GD53J01R) | 87 |
| 0.53 | D10-05 | JX898657 | *Uncultured soil fungus clone* | Ascomycota |  | 137 | \|  \| [GQ144684](http://www.ncbi.nlm.nih.gov/nucleotide/290760481?report=genbank&log$=nucltop&blast_rank=2&RID=SZ5N5RJB01R) \| \| --- \| --- \| | 82 |
| 0.53 | D11-10 | JX898659 | *Uncultured soil fungus clone* | Basidiomycota |  | 195 | [AY752989](http://www.ncbi.nlm.nih.gov/nucleotide/61187366?report=genbank&log$=nucltop&blast_rank=11&RID=T0V94ERA01R) | 88 |
| 0.53 | D11-16 | JX898662 | *Uncultured soil fungus clone* | Basidiomycota |  | 182 | [KC176336](http://www.ncbi.nlm.nih.gov/nucleotide/459256832?report=genbank&log$=nucltop&blast_rank=67&RID=T0VFNBGA01R) | 87 |
| 0.53 | D11-26 | JX898663 | *Uncultured soil fungus clone* | EDFL |  | 99 | \|  \| [KF750512](http://www.ncbi.nlm.nih.gov/nucleotide/575503354?report=genbank&log$=nucltop&blast_rank=2&RID=T0VJUD2B01R) \| \| --- \| --- \| | 88 |
| 0.53 | D11-29 | JX898664 | *Uncultured soil fungus clone* | EDFL |  | 388 | \|  \| [KF568432](http://www.ncbi.nlm.nih.gov/nucleotide/583834866?report=genbank&log$=nucltop&blast_rank=5&RID=T0VT3NR601R) \| \| --- \| --- \| | 58 |
| 0.53 | D11-37 | JX898666 | *Uncultured soil fungus clone* | EDFL |  | 368 | \|  \| [KF567069](http://www.ncbi.nlm.nih.gov/nucleotide/583833506?report=genbank&log$=nucltop&blast_rank=5&RID=T0VYY94D01R) \| \| --- \| --- \| | 55 |
| 0.53 | D12-27 | JX898674 | *Uncultured soil fungus clone* | [Glomeromycota](http://blast.st-va.ncbi.nlm.nih.gov/Blast.cgi#alnHdr_403390190) |  | 211 | [JN937441](http://www.ncbi.nlm.nih.gov/nucleotide/403390462?report=genbank&log$=nucltop&blast_rank=18&RID=T0W2ENB001R) | 90 |
| 0.53 | D12-32 | JX898675 | *Uncultured soil fungus clone* | Basidiomycota |  | 187 | [KF567090](http://www.ncbi.nlm.nih.gov/nucleotide/583833527?report=genbank&log$=nucltop&blast_rank=11&RID=T0W5MVV101R) | 87 |
| 0.53 | D02-40 | JX898641 | *Uncultured Calcarisporiella sp. clone* | EDFL |  | 407 | [AB617740](http://www.ncbi.nlm.nih.gov/nucleotide/359270139?report=genbank&log$=nucltop&blast_rank=3&RID=T0WB7HXV01R) | 88 |
| 0.53 | D01-39 | JX898638 | *Uncultured soil fungus clone* | EDFL |  | 99 | \|  \| [KF750512](http://www.ncbi.nlm.nih.gov/nucleotide/575503354?report=genbank&log$=nucltop&blast_rank=2&RID=T0VJUD2B01R) \| \| --- \| --- \| | 71 |
| 0.53 | D06-33 | JX898647 | *Uncultured soil fungus clone* | EDFL |  | 403 | \|  \| [AB617740](http://www.ncbi.nlm.nih.gov/nucleotide/359270139?report=genbank&log$=nucltop&blast_rank=4&RID=T0WNAD4V01R) \| \| --- \| --- \| | 87 |
| 0.53 | D07-26 | JX898649 | *Uncultured soil fungus clone* | EDFL |  | 399 | [KF568432](http://www.ncbi.nlm.nih.gov/nucleotide/583834866?report=genbank&log$=nucltop&blast_rank=5&RID=T0WSKYGV01R) | 50 |
| 0.53 | D06-13 | JX898644 | *Uncultured* [*Pseudogymnoascus sp.*](http://blast.st-va.ncbi.nlm.nih.gov/Blast.cgi#alnHdr_541136159) *clone* | Ascomycota |  | 538 | \|  \| [KF017871](http://www.ncbi.nlm.nih.gov/nucleotide/541136159?report=genbank&log$=nucltop&blast_rank=6&RID=T0WXYCYS01R) \| \| --- \| --- \| | 64 |
| 0.53 | D01-19 | JX534615 | *Uncultured soil fungus clone* | EDFL |  | 442 | [JX067943](http://www.ncbi.nlm.nih.gov/nucleotide/398802996?report=genbank&log$=nucltop&blast_rank=25&RID=T0X9FH5Z01R) | 88 |
| 0.53 | *D06-04 | JX534727 | *Uncultured* [*Mortierella exigua*](http://blast.st-va.ncbi.nlm.nih.gov/Blast.cgi#alnHdr_530253029) *clone* | EDFL |  | 1218 | \|  \| [KC009154](http://www.ncbi.nlm.nih.gov/nucleotide/530253029?report=genbank&log$=nucltop&blast_rank=5&RID=T0YCNUS301R) \| \| --- \| --- \| | 99 |
| 0.53 | D09-13 | JX534818 | *Uncultured* [*Mortierella zonata*](http://blast.st-va.ncbi.nlm.nih.gov/Blast.cgi#alnHdr_530253029) *clone* | EDFL |  | 1242 | \|  \| [KC018434](http://www.ncbi.nlm.nih.gov/nucleotide/511784205?report=genbank&log$=nucltop&blast_rank=12&RID=T0YZ8G3Y01R) \| \| --- \| --- \| | 99 |
| 0.53 | D05-38 | JX898642 | *Uncultured soil fungus clone* | EDFL |  | 243 | [EF681910](http://www.ncbi.nlm.nih.gov/nucleotide/197723529?report=genbank&log$=nucltop&blast_rank=9&RID=T0Z50VHG01R) | 85 |
| 0.53 | D06-02 | JX898643 | *Uncultured soil fungus clone* | EDFL |  | 207 | \|  \| [DQ393450](http://www.ncbi.nlm.nih.gov/nucleotide/87487501?report=genbank&log$=nucltop&blast_rank=16&RID=T0Z7ZA6G01R) \| \| --- \| --- \| | 86 |
| 0.53 | D10-06 | JX534843 | *Uncultured Hypochniciellum sp. clone* | Basidiomycota |  | 795 | \|  \| [AY586679](http://www.ncbi.nlm.nih.gov/nucleotide/46402602?report=genbank&log$=nucltop&blast_rank=17&RID=T0ZB6KTC01R) \| \| --- \| --- \| | 93 |
| 0.53 | D10-29 | JX534865 | *Uncultured Amylocorticiales sp. clone* | Basidiomycota |  | 1007 | [KC514898](http://www.ncbi.nlm.nih.gov/nucleotide/522358242?report=genbank&log$=nucltop&blast_rank=6&RID=T0ZJT66601R) | 99 |
| 0.53 | D12-08 | JX534910 | *Uncultured soil fungus clone* | EDFL |  | 1033 | \|  \| [FJ176706](http://www.ncbi.nlm.nih.gov/nucleotide/225216791?report=genbank&log$=nucltop&blast_rank=3&RID=T0ZR0XMB01R) \| \| --- \| --- \| | 95 |
| 0.53 | D12-28 | JX534923 | *Uncultured soil fungus clone* | EDFL |  | 771 | \|  \| [JX043247](http://www.ncbi.nlm.nih.gov/nucleotide/396581025?report=genbank&log$=nucltop&blast_rank=7&RID=T0ZW68JR01R) \| \| --- \| --- \| | 87 |
| 0.53 | D12-13 | JX534911 | *Uncultured soil fungus clone* | EDFL |  | 795 | \|  \| [JX043247](http://www.ncbi.nlm.nih.gov/nucleotide/396581025?report=genbank&log$=nucltop&blast_rank=7&RID=T101RKYE01R) \| \| --- \| --- \| | 88 |
| 0.53 | D12-14 | JX534912 | *Uncultured Hyphodontia sp. clone* | Basidiomycota |  | 985 | \|  \| [DQ340352](http://www.ncbi.nlm.nih.gov/nucleotide/92110620?report=genbank&log$=nucltop&blast_rank=5&RID=T103C64U01R) \| \| --- \| --- \| | 99 |
| 0.53 | D12-15 | JX534913 | *Uncultured soil fungus clone* | EDFL |  | 804 | [JX043247](http://www.ncbi.nlm.nih.gov/nucleotide/396581025?report=genbank&log$=nucltop&blast_rank=7&RID=T10B88PY01R) | 88 |
| 0.53 | D09-36 | JX534836 | [*Uncultured zygomycete clone*](http://blast.st-va.ncbi.nlm.nih.gov/Blast.cgi#alnHdr_170516523) | EDFL |  | 562 | \|  \| [EU490131](http://www.ncbi.nlm.nih.gov/nucleotide/170516523?report=genbank&log$=nucltop&blast_rank=5&RID=T10FTVM801R) \| \| --- \| --- \| | 82 |
| 0.53 | D12-20 | JX534916 | *Uncultured Phialophora sp. clone* | Ascomycota |  | 972 | AB190421 | 99 |
| 0.53 | D12-22 | JX534918 | *Uncultured Phialophora dancoi clone* | Ascomycota |  | 942 | AB190421 | 100 |
| 0.53 | D09-25 | JX534827 | [*Uncultured zygomycete clone*](http://blast.st-va.ncbi.nlm.nih.gov/Blast.cgi#alnHdr_170516523) | EDFL |  | 569 | [EU490131](http://www.ncbi.nlm.nih.gov/nucleotide/170516523?report=genbank&log$=nucltop&blast_rank=5&RID=T10RK9M601R) | 83 |
| 0.53 | D11-03 | JX534879 | [*Uncultured Ascomycota clone*](http://blast.st-va.ncbi.nlm.nih.gov/Blast.cgi#alnHdr_313482982) | Ascomycota |  | 953 | \|  \| [HQ432987](http://www.ncbi.nlm.nih.gov/nucleotide/313482982?report=genbank&log$=nucltop&blast_rank=3&RID=T10V479601R) \| \| --- \| --- \| | 98 |
| 0.53 | *D11-02 | JX534878 | *Uncultured* [*Ganoderma australe*](http://blast.st-va.ncbi.nlm.nih.gov/Blast.cgi#alnHdr_387273586) *clone* | Basidiomycota |  | 1005 | [JN048792](http://www.ncbi.nlm.nih.gov/nucleotide/387273586?report=genbank&log$=nucltop&blast_rank=2&RID=T11HU98U01R) | 100 |
| 0.53 | D11-06 | JX534882 | *Uncultured Ascomycota clone* | Ascomycota |  | 961 | [HQ432987](http://www.ncbi.nlm.nih.gov/nucleotide/313482982?report=genbank&log$=nucltop&blast_rank=2&RID=T10Z1J5J01R) | 99 |
| 0.53 | D11-12 | JX534886 | *Uncultured soil fungus clone* | Ascomycota |  | 931 | [HQ432987](http://www.ncbi.nlm.nih.gov/nucleotide/313482982?report=genbank&log$=nucltop&blast_rank=2&RID=T10Z1J5J01R) | 96 |
| 0.53 | D11-27 | JX534896 | *Uncultured* [*Ganoderma*](http://blast.st-va.ncbi.nlm.nih.gov/Blast.cgi#alnHdr_387273586) *sp. clone* | Basidiomycota |  | 992 | \|  \| [JN048792](http://www.ncbi.nlm.nih.gov/nucleotide/387273586?report=genbank&log$=nucltop&blast_rank=3&RID=T11CHGP401R) \| \| --- \| --- \| | 99 |
| 0.53 | D07-23 | JX534762 | *Uncultured Fusarium sp. clone* | Ascomycota |  | 918 | [EU860057](http://www.ncbi.nlm.nih.gov/nucleotide/218455080?report=genbank&log$=nucltop&blast_rank=5&RID=T1144UEZ01R) | 99 |
| 0.53 | D07-14 | JX534754 | *Uncultured soil fungus clone* | Ascomycota |  | 953 | [KF566557](http://www.ncbi.nlm.nih.gov/nucleotide/583832994?report=genbank&log$=nucltop&blast_rank=6&RID=T14KC28E01R) | 99 |
| 0.53 | D07-24 | JX534763 | *Uncultured soil fungus clone* | Ascomycota |  | 907 | [KC557359](http://www.ncbi.nlm.nih.gov/nucleotide/480553792?report=genbank&log$=nucltop&blast_rank=8&RID=T14NFSH401R) | 97 |
| 0.53 | D10-22 | JX534858 | *Uncultured Mortierella polycephala clone* | EDFL |  | 1223 | [JN939146](http://www.ncbi.nlm.nih.gov/nucleotide/358441415?report=genbank&log$=nucltop&blast_rank=2&RID=T118ME6801R) | 99 |
| 0.53 | D12-33 | JX534926 | *Uncultured Phialophora sp. clone* | Ascomycota |  | 937 | AB190421 | 98 |
| 0.53 | *D11-05 | JX534881 | *Uncultured* [*Ganoderma*](http://blast.st-va.ncbi.nlm.nih.gov/Blast.cgi#alnHdr_387273586) *sp. clone* | Basidiomycota |  | 987 | [JN048792](http://www.ncbi.nlm.nih.gov/nucleotide/387273586?report=genbank&log$=nucltop&blast_rank=3&RID=T11KAUCV01R) | 99 |
| 0.53 | D12-07 | JX534909 | *Uncultured Phialophora dancoi clone* | Ascomycota |  | 961 | \|  \| [AB190421](http://www.ncbi.nlm.nih.gov/nucleotide/307548415?report=genbank&log$=nucltop&blast_rank=2&RID=T11S8ST001R) \| \| --- \| --- \| | 99 |
| 0.53 | D03-07 | JX534650 | [*Uncultured Cercophora sp. clone*](http://blast.st-va.ncbi.nlm.nih.gov/Blast.cgi#alnHdr_408354657) | Ascomycota |  | 909 | [AY587937](http://www.ncbi.nlm.nih.gov/nucleotide/51104867?report=genbank&log$=nucltop&blast_rank=4&RID=T12EX48E01R) | 99 |
| 0.53 | D03-22 | JX534663 | *Uncultured Cercophora sparsa clone* | Ascomycota |  | 924 | [AY587937](http://www.ncbi.nlm.nih.gov/nucleotide/51104867?report=genbank&log$=nucltop&blast_rank=4&RID=T12EX48E01R) | 98 |
| 0.53 | D03-29 | JX534668 | *Uncultured Cercophora sparsa clone* | Ascomycota |  | 929 | [AY587937](http://www.ncbi.nlm.nih.gov/nucleotide/51104867?report=genbank&log$=nucltop&blast_rank=4&RID=T12EX48E01R) | 100 |
| 0.53 | D02-32 | JX534641 | *Uncultured Leuconeurospora sp. clone* | Ascomycota |  | 928 | \|  \| [FJ176884](http://www.ncbi.nlm.nih.gov/nucleotide/206598018?report=genbank&log$=nucltop&blast_rank=6&RID=T13DP1TP01R) \| \| --- \| --- \| | 99 |
| 0.53 | D12-09 | JX898668 | *Uncultured soil fungus clone* | EDFL |  | 551 | KF566381 | 90 |
| 0.53 | D07-40 | JX534775 | *Uncultured soil fungus clone* | Ascomycota |  | 826 | \|  \| [KF567483](http://www.ncbi.nlm.nih.gov/nucleotide/583833918?report=genbank&log$=nucltop&blast_rank=11&RID=T14RJBJX01R) \| \| --- \| --- \| | 94 |
| 1.06 | D07-01 | JX534742 | *Uncultured soil fungus clone* | Ascomycota |  | 985 | [KF566557](http://www.ncbi.nlm.nih.gov/nucleotide/583832994?report=genbank&log$=nucltop&blast_rank=6&RID=T14ET5H601R) | 99 |
| 1.06 | D12-12 | JX898771 | *Uncultured soil fungus clone* | [Glomeromycota](http://blast.st-va.ncbi.nlm.nih.gov/Blast.cgi#alnHdr_403390190) |  | 545 | [KF566381](http://www.ncbi.nlm.nih.gov/nucleotide/583832818?report=genbank&log$=nucltop&blast_rank=10&RID=T13M47ZG01R) | 99 |
| 1.06 | D12-19 | JX898773 | *Uncultured soil fungus clone* | EDFL |  | 556 | \|  \| [KF566381](http://www.ncbi.nlm.nih.gov/nucleotide/583832818?report=genbank&log$=nucltop&blast_rank=10&RID=T13RE5D201R) \| \| --- \| --- \| | 91 |
| 1.06 | D03-15 | JX534658 | *Uncultured Mortierella indohii clone* | EDFL |  | 1194 | [EU688966](http://www.ncbi.nlm.nih.gov/nucleotide/189031489?report=genbank&log$=nucltop&blast_rank=3&RID=T1707TH501R) | 99 |
| 1.06 | D03-18 | JX534660 | *Uncultured Tetracladium sp. clone* | Ascomycota |  | 935 | \|  \| [KF768462](http://www.ncbi.nlm.nih.gov/nucleotide/559098697?report=genbank&log$=nucltop&blast_rank=4&RID=T172ZG6B01R) \| \| --- \| --- \| | 99 |
| 1.06 | D12-06 | JX534908 | *Uncultured Cladosporium coralloides clone* | Ascomycota |  | 965 | \|  \| [KF611802](http://www.ncbi.nlm.nih.gov/nucleotide/540360775?report=genbank&log$=nucltop&blast_rank=6&RID=T177372H01R) \| \| --- \| --- \| | 99 |
| 1.06 | D10-15 | JX534852 | *Uncultured Uncinocarpus reesii clone* | Ascomycota |  | 880 | \|  \| [JQ434632](http://www.ncbi.nlm.nih.gov/nucleotide/379142203?report=genbank&log$=nucltop&blast_rank=7&RID=T17C1SBA01R) \| \| --- \| --- \| | 98 |
| 1.06 | D03-10 | JX534653 | *Uncultured Fusarium sp. clone* | Ascomycota |  | 887 | [GU055596](http://www.ncbi.nlm.nih.gov/nucleotide/261871842?report=genbank&log$=nucltop&blast_rank=4&RID=T17FZA4T01R) | 100 |
| 1.06 | D10-27 | JX534863 | *Uncultured Mortierella polycephala clone* | EDFL |  | 1223 | [JN939145](http://www.ncbi.nlm.nih.gov/nucleotide/358441414?report=genbank&log$=nucltop&blast_rank=2&RID=T17JZ3EN01R) | 99 |
| 1.06 | D01-20 | JX534616 | *Uncultured Leuconeurospora sp. clone* | Ascomycota |  | 957 | [FJ176884](http://www.ncbi.nlm.nih.gov/nucleotide/206598018?report=genbank&log$=nucltop&blast_rank=2&RID=T17PJ76A01R) | 100 |
| 1.59 | D12-18 | JX534915 | *Uncultured soil fungus clone* | EDFL |  | 977 | \|  \| [EU516968](http://www.ncbi.nlm.nih.gov/nucleotide/187961890?report=genbank&log$=nucltop&blast_rank=4&RID=T17W2UPA01R) \| \| --- \| --- \| | 94 |
| 2.12 | D09-12 | JX534817 | *Uncultured soil fungus clone* | EDFL |  | 1083 | [EU861720](http://www.ncbi.nlm.nih.gov/nucleotide/194475313?report=genbank&log$=nucltop&blast_rank=6&RID=T1809ZKY01R) | 92 |
| 2.65 | D01-06 | JX898636 | *Uncultured soil fungus clone* | Basidiomycota |  | 414 | \|  \| [JX898661](http://www.ncbi.nlm.nih.gov/nucleotide/511488266?report=genbank&log$=nucltop&blast_rank=8&RID=T185MHSS01R) \| \| --- \| --- \| | 80 |
| 2.65 | D06-06 | JX534728 | *Uncultured soil fungus clone* | EDFL |  | 1151 | [KF566486](http://www.ncbi.nlm.nih.gov/nucleotide/583832923?report=genbank&log$=nucltop&blast_rank=7&RID=T18A2R7P01R) | 97 |
| 3.18 | D01-31 | JX534622 | *Uncultured soil fungus clone* | EDFL |  | 1201 | [KF565913](http://www.ncbi.nlm.nih.gov/nucleotide/583832350?report=genbank&log$=nucltop&blast_rank=9&RID=T18D7UFY01R) | 97 |
| 3.71 | D02-06 | JX534631 | *Uncultured Sphaerosporium equinum clone* | Ascomycota |  | 950 | [JQ434638](http://www.ncbi.nlm.nih.gov/nucleotide/379142209?report=genbank&log$=nucltop&blast_rank=2&RID=T18HKNRC01R) | 100 |
| 3.71 | D11-07 | JX534883 | *Uncultured Oxyporus corticola clone* | Basidiomycota |  | 1022 | \|  \| [KC176679](http://www.ncbi.nlm.nih.gov/nucleotide/452887918?report=genbank&log$=nucltop&blast_rank=3&RID=T18NZAN201R) \| \| --- \| --- \| | 100 |
| 3.71 | D02-28 | JX534640 | *Uncultured Mucor flavus clone* | EDFL |  | 1127 | \|  \| [EU071390](http://www.ncbi.nlm.nih.gov/nucleotide/156523894?report=genbank&log$=nucltop&blast_rank=2&RID=T18U6EDB01R) \| \| --- \| --- \| | 99 |
| 4.24 | D07-03 | JX534744 | *Uncultured Cephalotheca sulfurea clone* | Ascomycota |  | 948 | \|  \| [FJ808681](http://www.ncbi.nlm.nih.gov/nucleotide/258640202?report=genbank&log$=nucltop&blast_rank=3&RID=T1939PAB01R) \| \| --- \| --- \| | 99 |
| 4.24 | D08-06 | JX534780 | *Uncultured soil fungus clone* | EDFL |  | 848 | [KF565875](http://www.ncbi.nlm.nih.gov/nucleotide/583832312?report=genbank&log$=nucltop&blast_rank=11&RID=T197E0J201R) | 95 |
| 5.3 | D02-36 | JX534642 | *Uncultured* [*Mortierella elongata*](http://blast.st-va.ncbi.nlm.nih.gov/Blast.cgi#alnHdr_223929948) *clone* | EDFL |  | 1216 | \|  \| [FJ161938](http://www.ncbi.nlm.nih.gov/nucleotide/223929948?report=genbank&log$=nucltop&blast_rank=21&RID=T19BEZCA01R) \| \| --- \| --- \| | 99 |
| 5.83 | D06-03 | JX534726 | [*Uncultured Pichia sp. clone*](http://blast.st-va.ncbi.nlm.nih.gov/Blast.cgi#alnHdr_408354785) | Ascomycota |  | 918 | \|  \| [HM036077](http://www.ncbi.nlm.nih.gov/nucleotide/296248921?report=genbank&log$=nucltop&blast_rank=11&RID=T19G6NGP01R) \| \| --- \| --- \| | 97 |
| 7.42 | *D05-02 | JX534699 | *Uncultured Chaetomidium arxii clone* | Ascomycota |  | 852 | [JX280740](http://www.ncbi.nlm.nih.gov/nucleotide/475644241?report=genbank&log$=nucltop&blast_rank=17&RID=T19MWED701R) | 98 |
| 7.95 | *D03-11 | JX534654 | *Uncultured Mortierella indohii clone* | EDFL |  | 1190 | \|  \| [EU736318](http://www.ncbi.nlm.nih.gov/nucleotide/209402438?report=genbank&log$=nucltop&blast_rank=16&RID=T19TXR3N01R) \| \| --- \| --- \| | 99 |
| 8.48 | D01-05 | JX534606 | *Uncultured soil fungus clone* | EDFL |  | 468 | [GU376405](http://www.ncbi.nlm.nih.gov/nucleotide/289431759?report=genbank&log$=nucltop&blast_rank=23&RID=T19X9WWS01R) | 90 |
| 8.48 | D01-15 | JX534612 | *Uncultured Doratomyces sp.clone* | Ascomycota |  | 955 | KC009280 | 100 |
| 9.01 | *D01-18 | JX534614 | *Uncultured Geomyces pannorum clone* | Ascomycota |  | 957 | [JQ768405](http://www.ncbi.nlm.nih.gov/nucleotide/391883734?report=genbank&log$=nucltop&blast_rank=12&RID=T1A62KZR01R) | 100 |
| 14.31 | *D03-05 | JX534648 | *Uncultured Trichosporon dulcitum clone* | Basidiomycota |  | 959 | [JN939493](http://www.ncbi.nlm.nih.gov/nucleotide/358441577?report=genbank&log$=nucltop&blast_rank=15&RID=T1A9MUCS01R) | 100 |

^a^Relative abundance for the combined libraries, which was used to sort the entries

^b^OTUs were characterized by Mothur program [1], the OTU is ≥97% similar to a fungal isolate

^c^BLASTN [2] score value

^d^Accession number of the closest database match

^e^Level of similarity for pairwise alignments with the closest match, using the Martinez-Needleman-Wunsch algorithm [2]

^*^Common OTUs recovered from LSU and ITS cloned libraries
