# Supplementary material for: Mycobiome of the Bat White Nose Syndrome Affected Caves and Mines Reveals Diversity of Fungi and Local Adaptation by the Fungal Pathogen Pseudogymnoascus (Geomyces) destructans
Source: PLoS One. 2014 Sep 29;9(9):e108714. doi: 10.1371/journal.pone.0108714 (PMC4181696; doi:10.1371/journal.pone.0108714)
Supplement: Table S9 — List of common OTUs identified by CD and CI methods. (DOCX) [file pone.0108714.s010.docx]

Table S9. List of common OTUs identified by CD and CI methods

^a^ One representative strain (from the CD method) and one representative clone (from the CI method) is presented for the 13 shared OTUs. ^b^ Abundance indicates the number of strains or clones for each OTU in the entire data set. ^c^ BLASTN score. ^d^ Accession number of the closest database match. EDFL, early diverging fungal lineages.
