# Supplementary material for: Mycobiome of the Bat White Nose Syndrome Affected Caves and Mines Reveals Diversity of Fungi and Local Adaptation by the Fungal Pathogen Pseudogymnoascus (Geomyces) destructans
Source: PLoS One. 2014 Sep 29;9(9):e108714. doi: 10.1371/journal.pone.0108714 (PMC4181696; doi:10.1371/journal.pone.0108714)
Supplement: Table S10 — Alpha diversity analyses of fungal DNA clones recovered in the WNS-infested environmental samples. (DOCX) [file pone.0108714.s011.docx]

Table S10. Alpha diversity analyses of fungal DNA clones recovered in the WNS-infested environmental samples.

| **Sample ID** | **Reads** | **Cut off = 0.03** | | | | | |
| --- | --- | --- | --- | --- | --- | --- | --- |
|  |  | **OTU** | **ACE** | **Chao-1** | **Coverage** | **Shannon** | **Simpson** |
| LSU | 353 | 189 | 938 (783, 1134) | 595 (430, 873) | 0.611898 | 4.86 (4.75, 4.98) | 0.0101 (0.0071, 0.013) |
| ITS | 241 | 73 | 394  (298, 530) | 237 (140, 466) | 0.796680 | 3.04 (2.83, 3.25) | 0.115 (0.0874, 0.1427) |

Note: LSU, large-subunit rDNA; ITS, internal transcribed spacer rDNA.
